# Supplementary material for: The structural variation landscape in 492 Atlantic salmon genomes
Source: Nat Commun. 2020 Oct 14;11:5176. doi: 10.1038/s41467-020-18972-x (PMC7560756; doi:10.1038/s41467-020-18972-x)
Supplement: Supplementary file 1 — Supplementary Information [file 41467_2020_18972_MOESM1_ESM.pdf]

Supplementary Information

The structural variation landscape in 492 Atlantic salmon genomes

Alicia C. Bertolotti, Ryan M. Layer, Manu Kumar Gundappa, Michael D. Gallagher, Ege Pehlivanoglu, Torfinn Nome, Diego Robledo, Matthew P. Kent, Line L. Røsæg, Matilde M. Holen, Teshome D. Mulugeta, Thomas J. Ashton, Kjetil Hindar, Harald Sægrov, Bjørn Florø-Larsen, Jaakko Erkinaro, Craig R. Primmer, Louis Bernatchez, Samuel A.M. Martin, Ian A. Johnston, Simen R. Sandve, Sigbjørn Lien, Daniel J. Macqueen

Contents:

Supplementary Figure 1. Snakemake pipeline for end-to-end SV detection.
Supplementary Figure 2. Locations of complex regions in Atlantic salmon genome.
Supplementary Figure 3. Example of SV-plaudit image for a high confidence deletion SV call
Supplementary Figure 4. Example of SV-plaudit image for a false positive deletion SV call excluded from further analyses.
Supplementary Figure 5. Example of SV-plaudit image for a high confidence duplication SV call retained in further analyses
Supplementary Figure 6. Example of SV-plaudit image for a false positive duplication SV call excluded from further analyses
Supplementary Figure 7. Example of SV-plaudit image for a high confidence inversion SV call retained in further analyses
Supplementary Figure 8. Example of SV-plaudit image for a false positive inversion SV call excluded from further analyses
Supplementary Figure 9. SV sizes before SV-plaudit curation
Supplementary Figure 10. Sequencing depth was not a strong predictor of the final number of high-confidence SVs retained after SV-plaudit curation.
Supplementary Figure 11. Summary of 168 SV regions used for MinION amplicon sequencing to validate Lumpy/SVtyper SV and genotype calls.
Supplementary Figure 12. Example of congruence between SV/genotype calls and data generated by MinION amplicon sequencing.
Supplementary Figure 13. Example of congruence between SV/genotype calls and data generated by MinION amplicon sequencing.
Supplementary Figure 14. Example of congruence between SV/genotype calls and data generated by MinION amplicon sequencing.
Supplementary Figure 15. PCAs showing the same data presented in Fig. 1g-i (main text), except visualized according to latitude
Supplementary Figure 16. PCA analyses done on SV genotype calls prior to SV-plaudit curation
Supplementary Figure 17. SV annotation by SnpEff
Supplementary Figure 18. Overlap between high confidence SVs and protein coding genes in the ICSASG_v2 annotation.
Supplementary Figure 19. Number of high impact annotations per snpEff effect for high-confidence Atlantic salmon SVs.
Supplementary Figure 20. Maximum likelihood tree presented in Fig. 2 including sample identifiers, genomic locations of pTSsa2 sequences and bootstrap values.
Supplementary Figure 21. Circos plot showing the genomic locations of pTSsa2 sequences in the Atlantic salmon genome
Supplementary Figure 22. Expression characteristics of ohnologs depending on SV overlap.
Supplementary Figure 23. Tissue expression levels comparing SV outliers with transcriptome wide expectations for nine tissues
Supplementary Figure 24. Tissue specificity comparing SV outliers with transcriptome wide expectations for nine tissues

Supplementary Figure 25. Heatmap showing individual SV genotypes for 45 SV outliers linked to synapse genes.

Supplementary Note 1. Python script used to extract gap regions in the ICSASG_v2 genome and and convert the outputs to a BED file
Supplementary Note 2. Snakefiles and associated code for SV calling pipeline
Supplementary Note 3: Custom R script used to obtain Fst values from random comparisons and establish probability value for outlier SVs

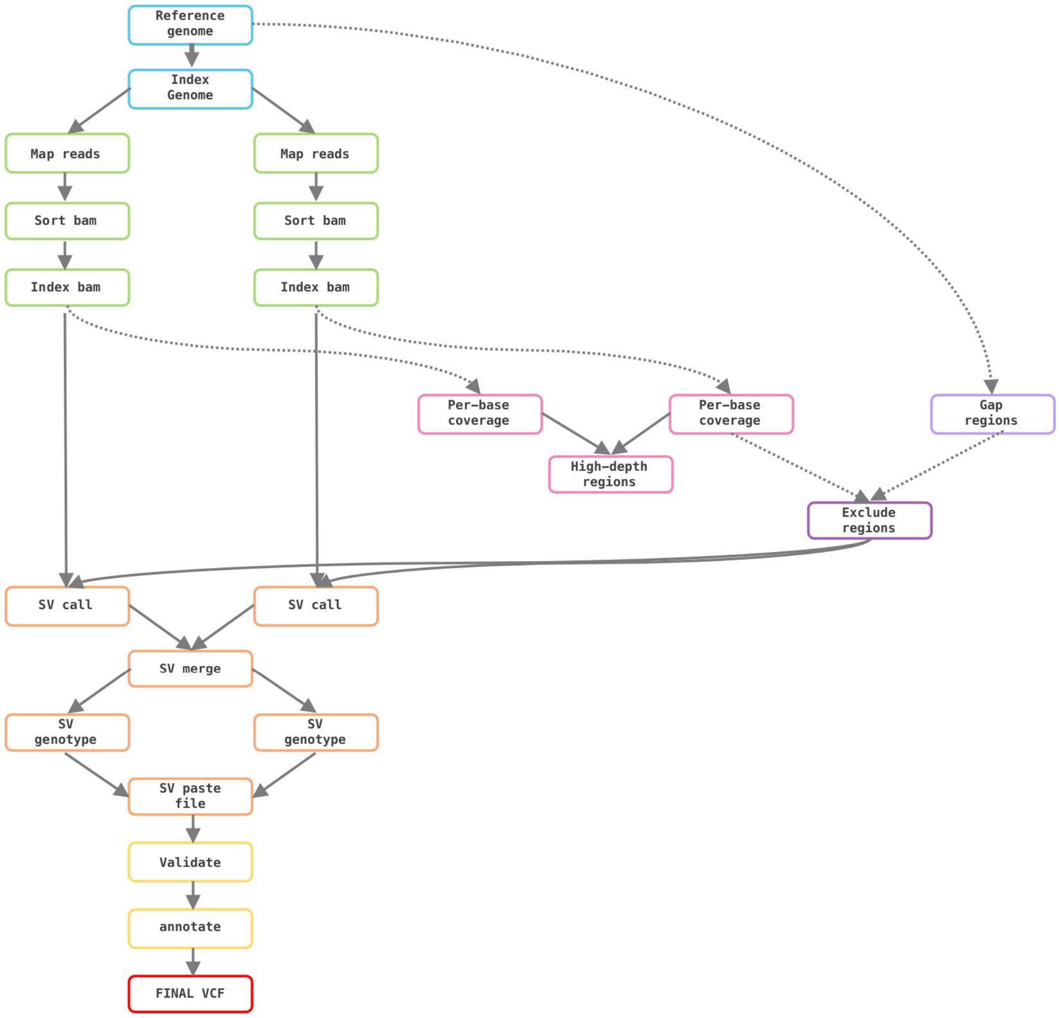

Supplementary Figure 1. Snakemake (v.3.11.0) pipeline for SV detection. The pipeline has the steps to go from raw FASTQ reads to a finalized VCF file with annotated SV calls/genotypes across all samples in a study. Hashed arrows indicate steps that need to be taken once per reference genome. On the left side of the figure, two parallel tracks are shown to indicate the steps performed independently in different samples (i.e. 'Map reads', 'Sort bam', 'Index bam', 'SV call' and 'SV genotype'), as opposed to steps performed on data combined across different samples. Respecting Snakemake style, a template command list was written into a Snakefile, where the global rule ('all rule') set was to produce one output with all samples and SVs identified (provided in Supplementary Note 2). Conda environments were created with Python v.2.7 and v.3, given that Lumpy Smoove was written in Python v.2.7 and Snakemake was written in Python v.3 (see Supplementary Note 2).

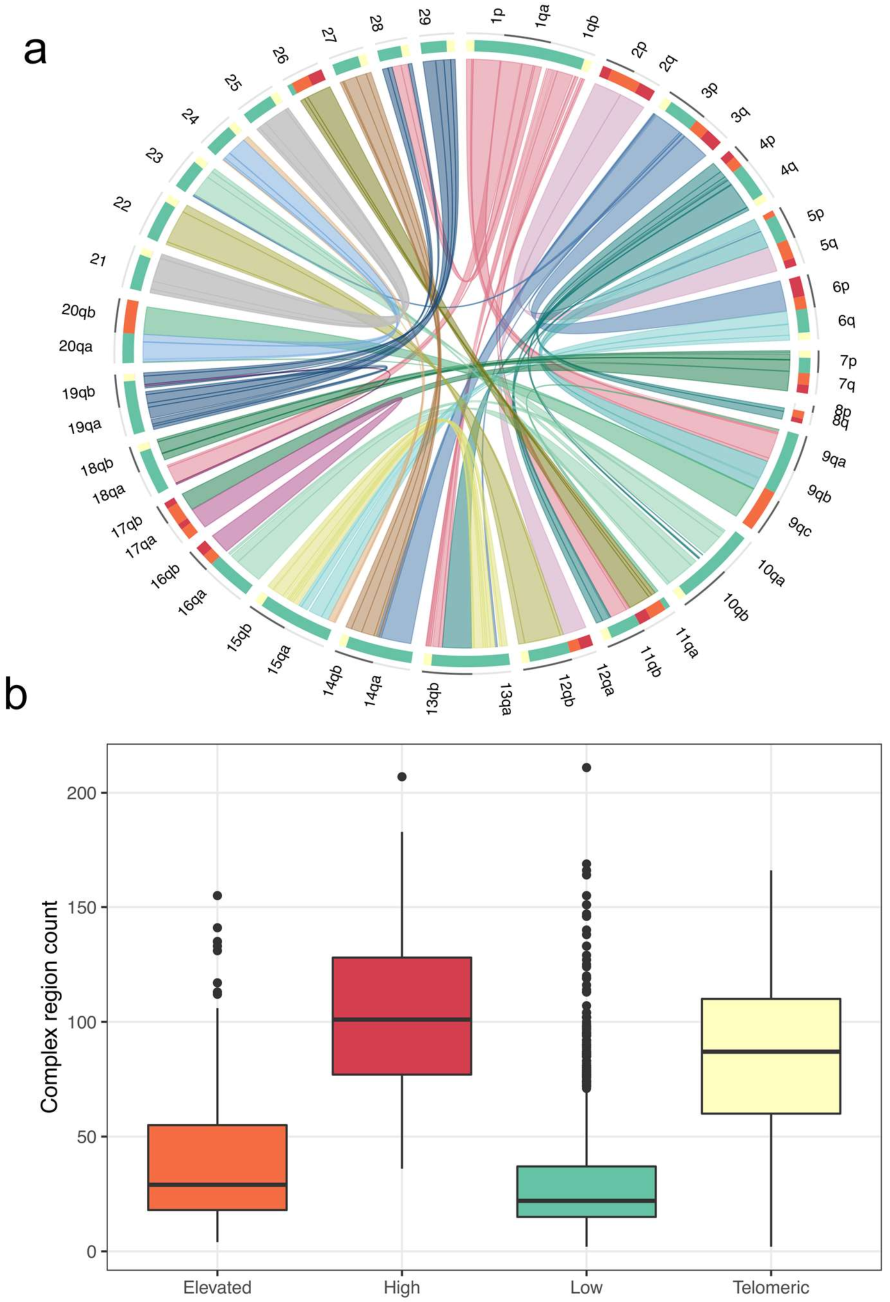

Supplementary Figure 2. Locations of complex regions in Atlantic salmon genome. **a** Circos plot adapted from [17], showing the 29 Atlantic salmon chromosomes divided into duplicated regions retained from the Ss4R WGD event (linked by inner circle ribbons). The main track shows each chromosome arm (names follow Atlantic salmon nomenclature [17]) divided into four categories: 'telomeres' (yellow), duplicated regions of 'low' duplicated similarity (~87% - green), duplicated regions of 'elevated' duplicated similarity (90-95% - orange) and duplicated regions of 'high' duplicated similarity (>95% - red). **b** Boxplot of counts of complex regions (defined as gap regions and regions with coverage ≥100x) across the four different categories of the genome. Complex regions were associated with an exceptionally high rate of false positive SV calls and filtered from the analysis (see Main text). Definition of box and whisker plots: the box spans the interquartile range, with the median (Q2) as a central bar, and respective upper and lower bounds representing the minimum and maximum values within the 25th percentile (Q1) and 75th percentile (Q3). The bounds of the upper and lower whisker are the largest and smallest values that lie within 1.5 times above Q3 and below Q1, respectively. Outliers out with these bounds are shown as individual points.

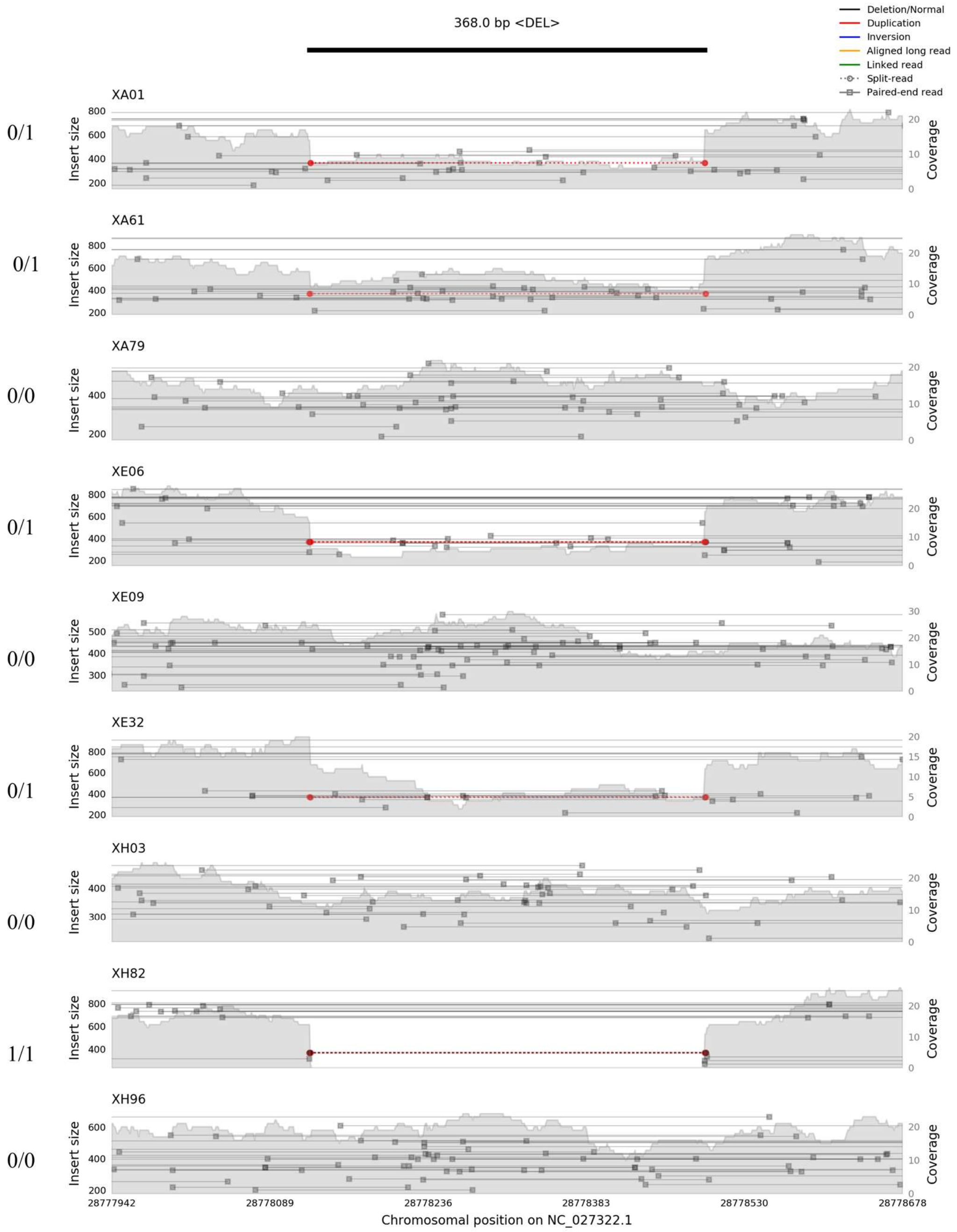

Supplementary Figure 3. Example of SV-plaudit image for a high confidence deletion SV call. Nine fish are shown with genotype calls given at the left of each samplot image. Also provided is the estimated breakpoint and size of the predicted deletion (black line at top of image), and a key describing the visualized evidence underlying the SV call.

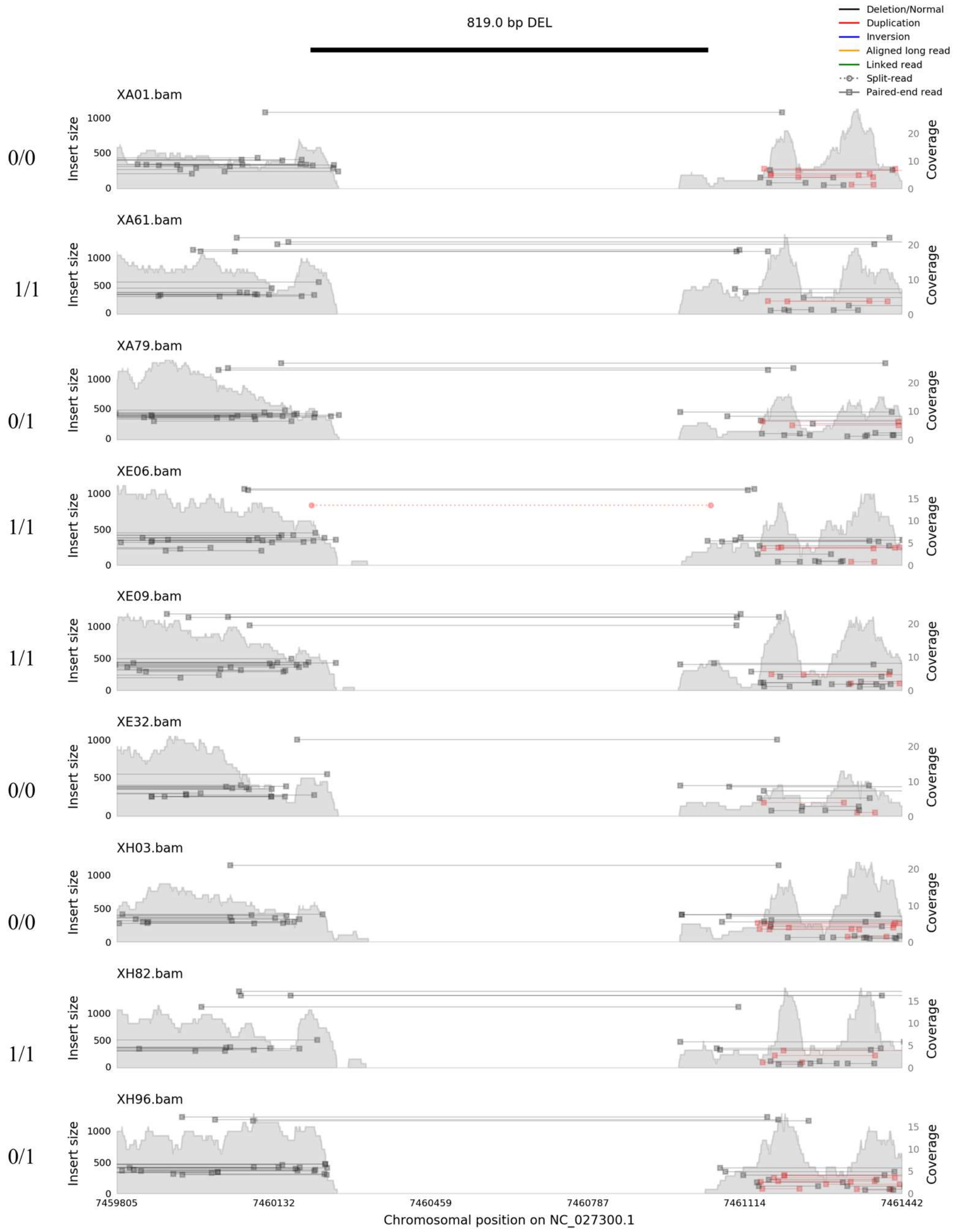

Supplementary Figure 4. Example of SV-plaudit image for a false positive deletion SV call excluded from further analyses. Nine fish are shown with genotype calls given at the left of each samplot image. Also provided is the estimated breakpoint and size of the predicted deletion (black line at top of image), and a key describing the visualized evidence underlying the SV call.

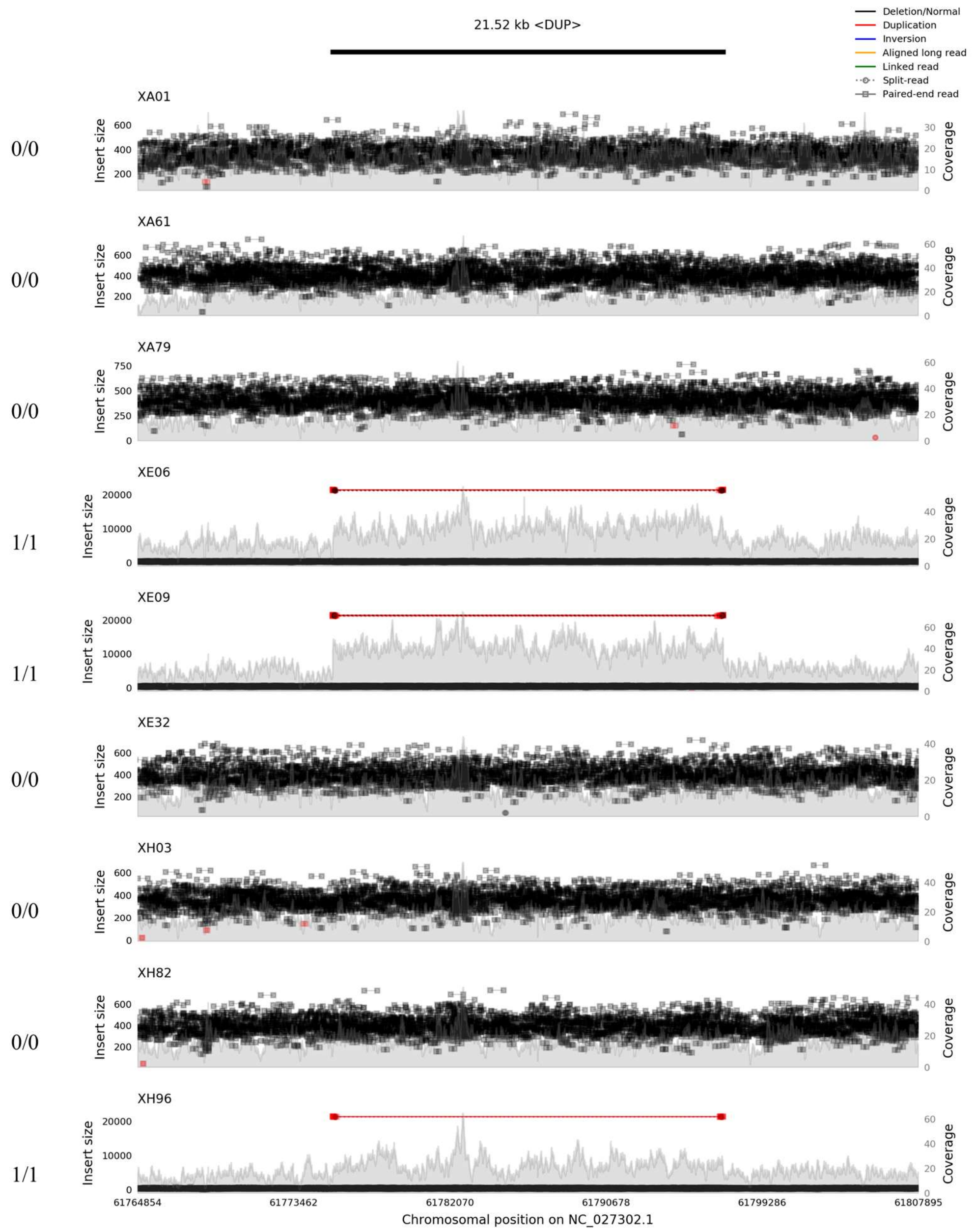

Supplementary Figure 5. Example of SV-plaudit image for a high confidence duplication SV call retained in further analyses. Nine fish are shown with genotype calls given at the left of each samplot image. Also provided is the estimated breakpoint and size of the predicted deletion (black line at top of image), and a key describing the visualized evidence underlying the SV call.

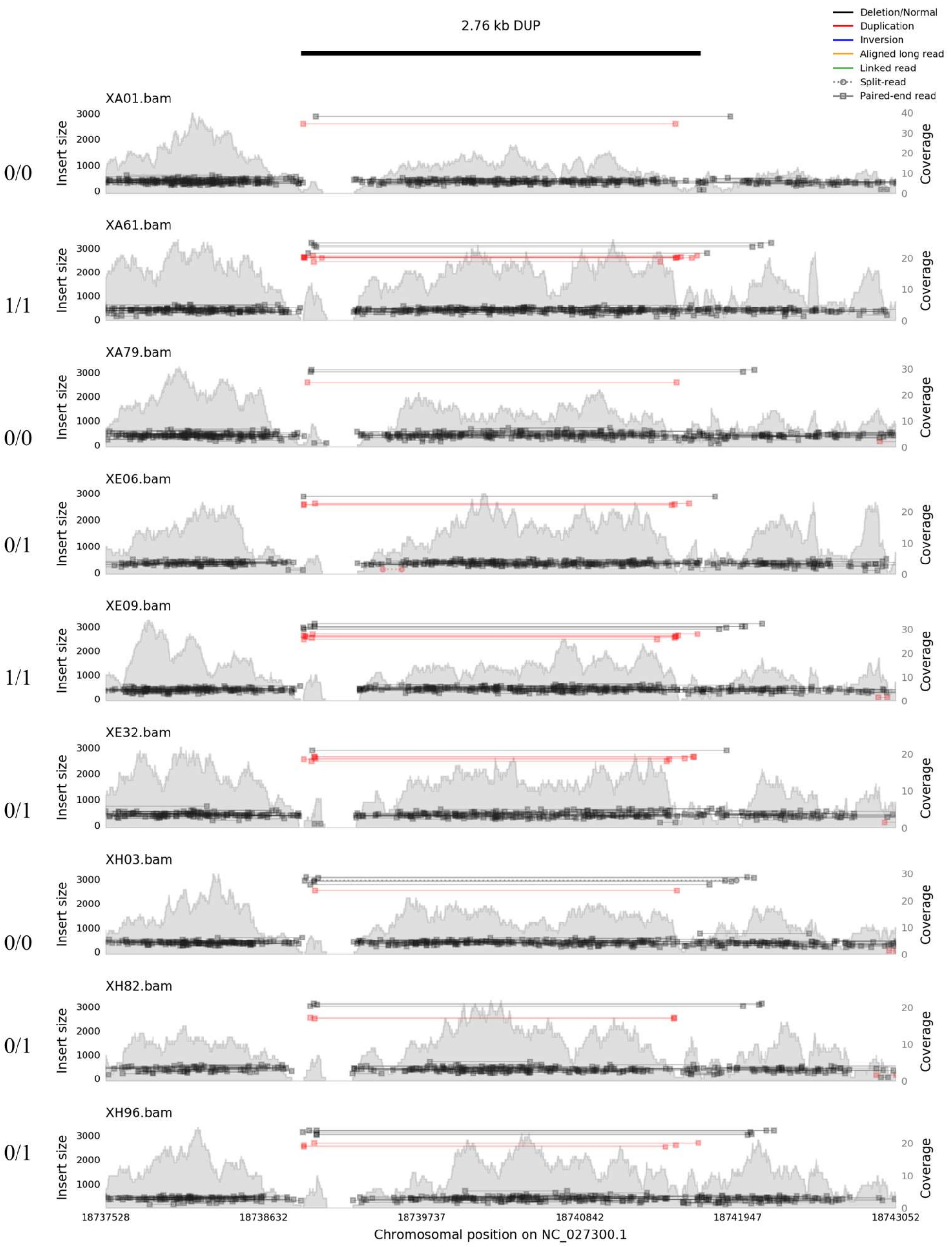

Supplementary Figure 6. Example of SV-plaudit image for a false positive duplication SV call excluded from further analyses. Nine fish are shown with genotype calls given at the left of each samplot image. Also provided is the estimated breakpoint and size of the predicted deletion (black line at top of image), and a key describing the visualized evidence underlying the SV call.

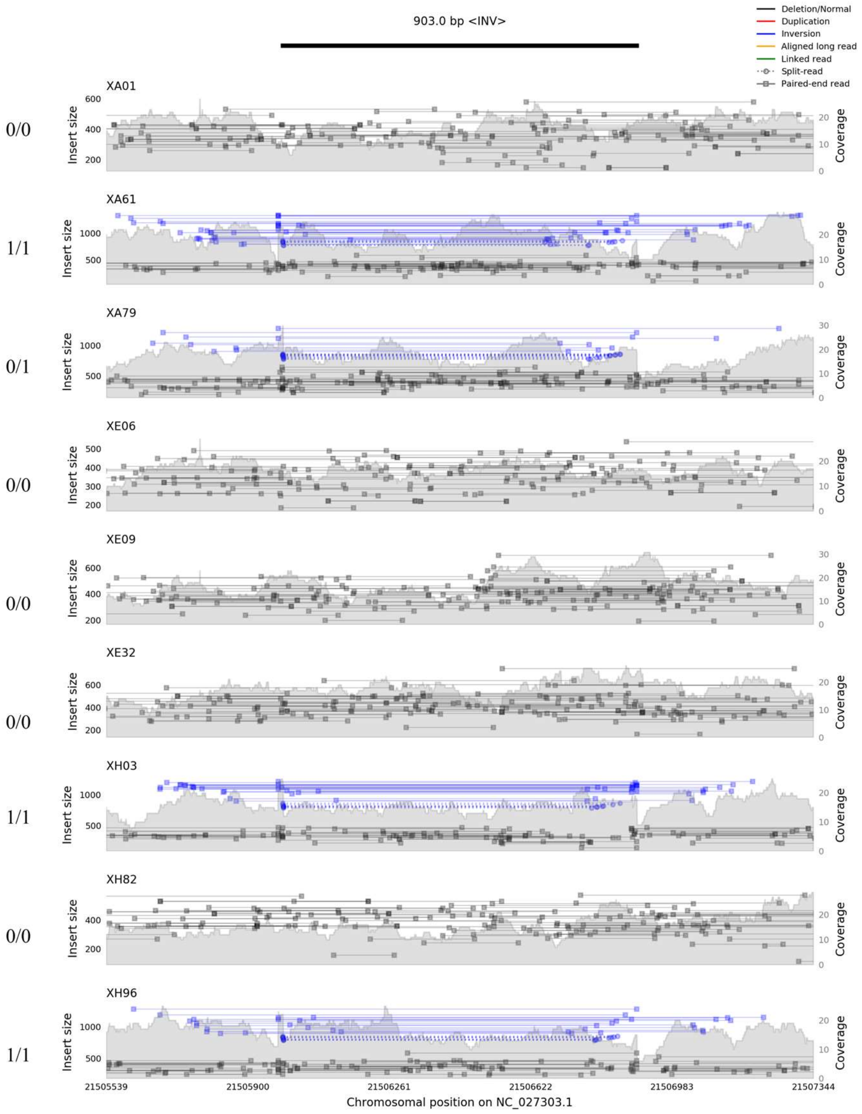

Supplementary Figure 7. Example of SV-plaudit image for a high confidence inversion SV call retained in further analyses. Nine fish are shown with genotype calls given at the left of each samplot image. Also provided is the estimated breakpoint and size of the predicted deletion (black line at top of image), and a key describing the visualized evidence underlying the SV call.

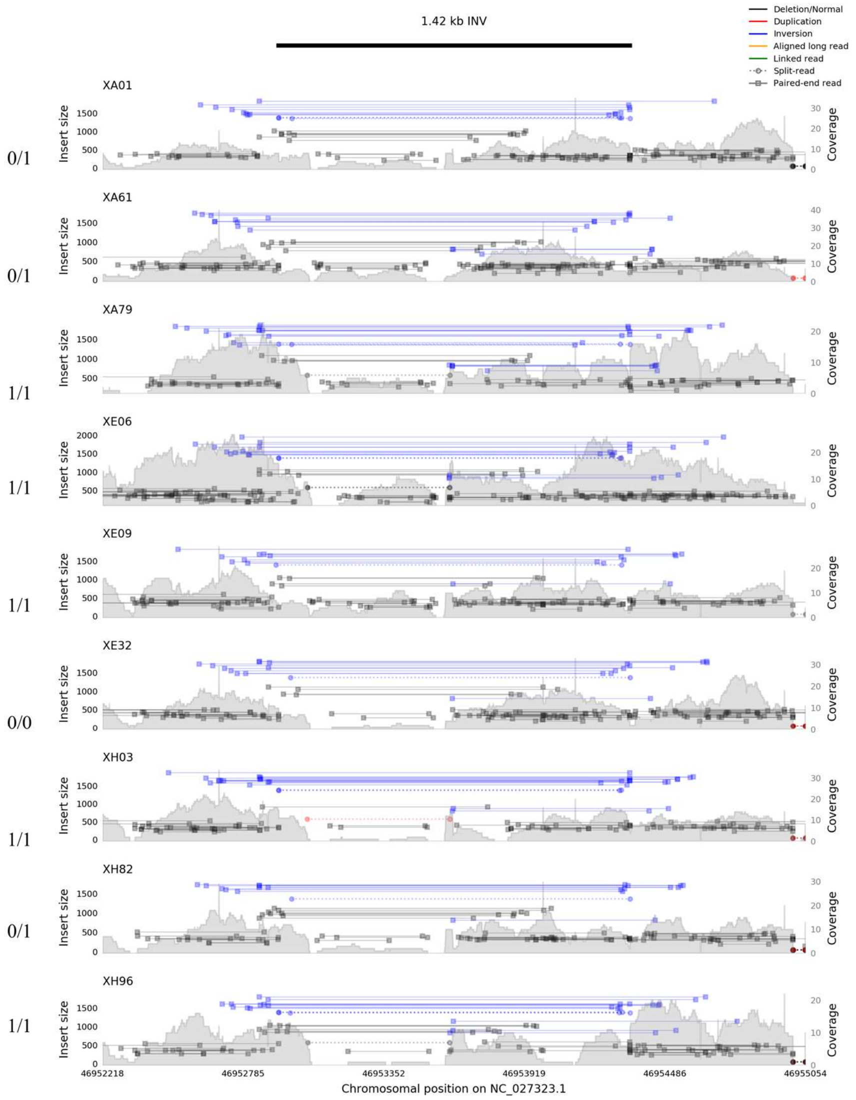

Supplementary Figure 8. Example of SV-plaudit image for a false positive inversion SV call excluded from further analyses. Nine fish are shown with genotype calls given at the left of each samplot image. Also provided is the estimated breakpoint and size of the predicted deletion (black line at top of image), and a key describing the visualized evidence underlying the SV call.

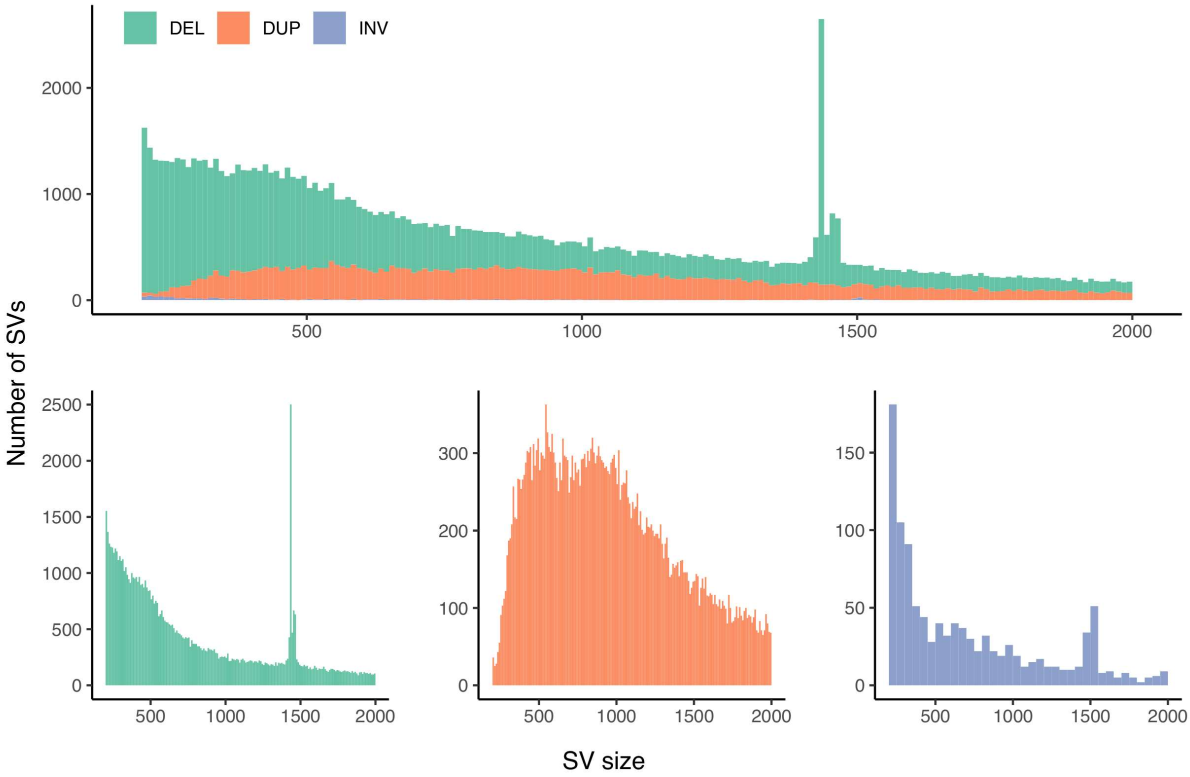

Supplementary Figure 9. SV sizes before filtering and SV-plaudit curation. Note the peak observed in the deletion calls, which remained after SV-plaudit curation, and is further characterized in Fig. 2.

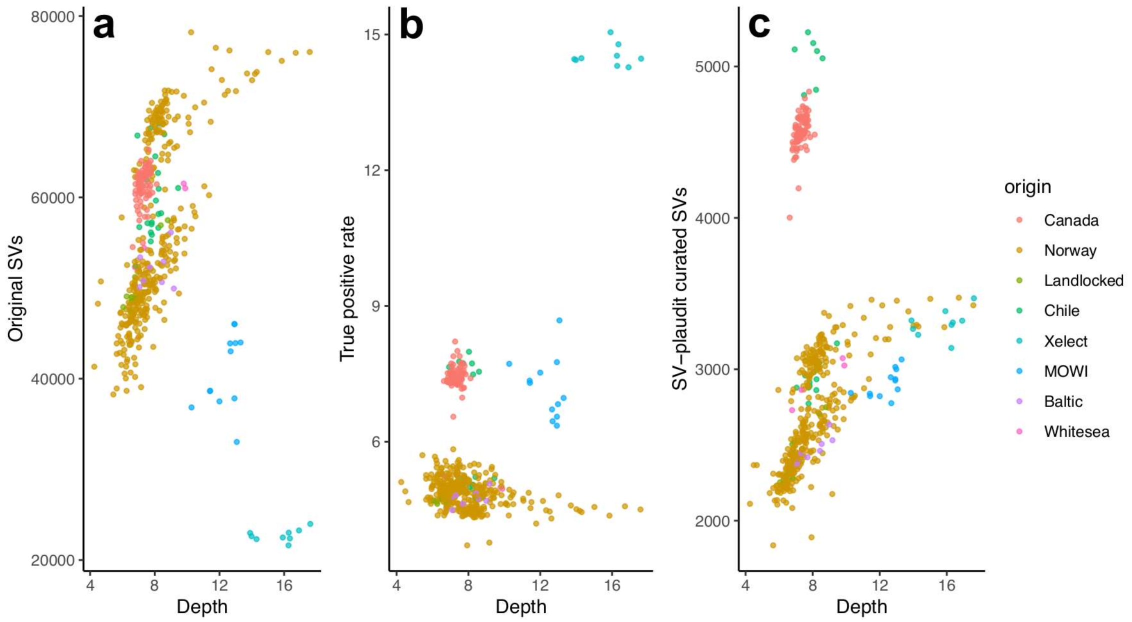

Supplementary Figure 10. Sequencing depth was not a strong predictor of the final number of high-confidence SVs retained after SV-plaudit curation. The scatterplots show sequencing depth plotted against **a** the number of original/unfiltered SV calls, **b** the true positive rate (%) for SV calls (number of retained high confidence SV calls after SV-plaudit validation / number of original SV calls) and **c** the final number of SV-plaudit curated SV calls. The origin of each sample is shown by a different colour according to the given key (see Supplementary Data 1). Our hypothesis was that increased sequencing depth would lead to a lower FDR, along with an increased true positive rate. However, there was no significant correlation between sequencing depth and the number of unfiltered SVs (part **a**, Pearson's correlation coefficient = 0.07, P = 0.11) and a weak but significant positive correlation between sequencing depth and true positive SV call rate (part **b**; Pearson's correlation coefficient = 0.34, $P < 0.001$). Observing the final SV-plaudit curated data (part **c**), it can be seen that a few samples skew the expected relationship, i.e. either have relatively high sequencing depth with a relatively high number of unfiltered SVs and relatively low true positive SV call rate. It can also be seen that most the variability is explained by study differences; the data was derived from five different studies, which include variation not just in sequencing depth, but also biology (i.e. distinct geographical locations, farmed vs. wild fish), as well as technological factors (four distinct sequencing platforms; many different labs involved in DNA extraction/library preps). Together, these features may obscure the expected relationship between SV detection and sequencing depth.

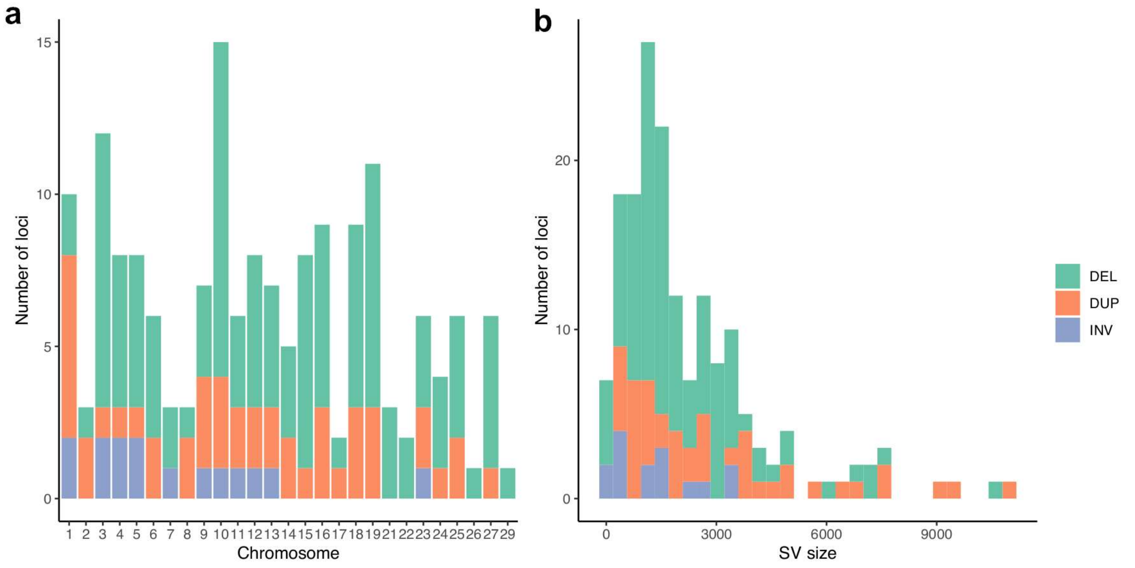

Supplementary Figure 11. Summary of 168 SV regions used for MinION amplicon sequencing to validate Lumpy/SVtyper SV and genotype calls. **a** Number of SV regions targeted on different salmon chromosomes. **b** Size distribution of SV regions targeted by PCR.

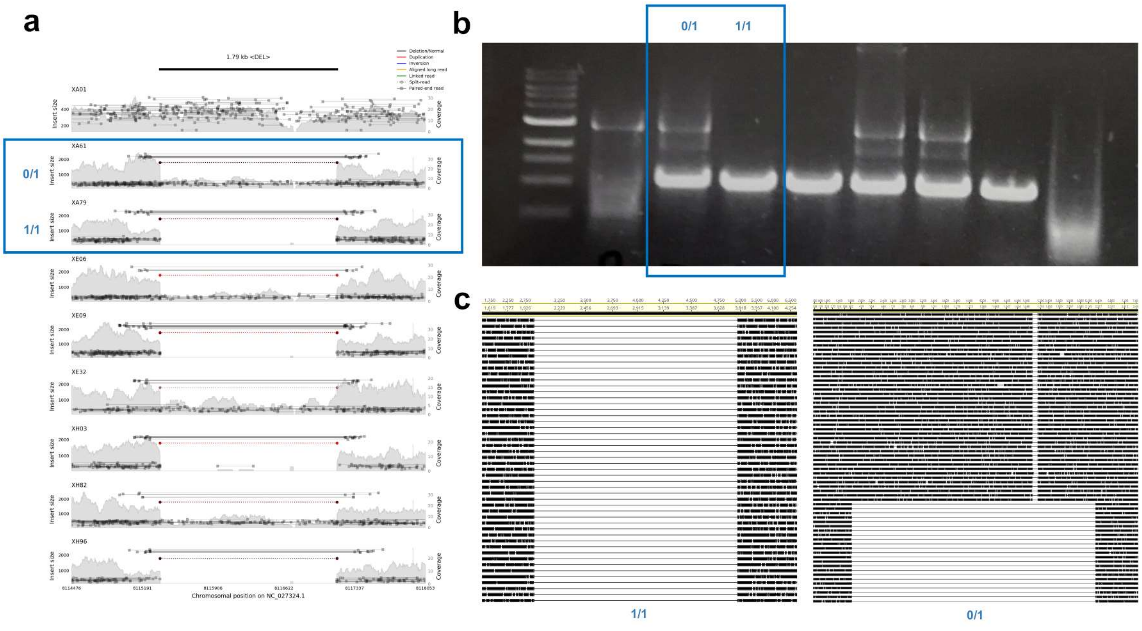

Supplementary Figure 12. Example of congruence between SV/genotype calls and data generated by MinION amplicon sequencing. **a** SV-plaudit deletion plot for 9 tested salmon individuals. Circled in blue are one 0/1 genotype call and one 1/1 genotype call. **b** agarose gel image for the same individuals, with two calls highlighted in the gel image, showing the same genotypes. The DNA ladder in the left lane is a 1 kb DNA Ladder from New England Biolabs (Catalog# N3232S). **c** Alignments of amplicons sequenced for the samples highlighted in part **b**.

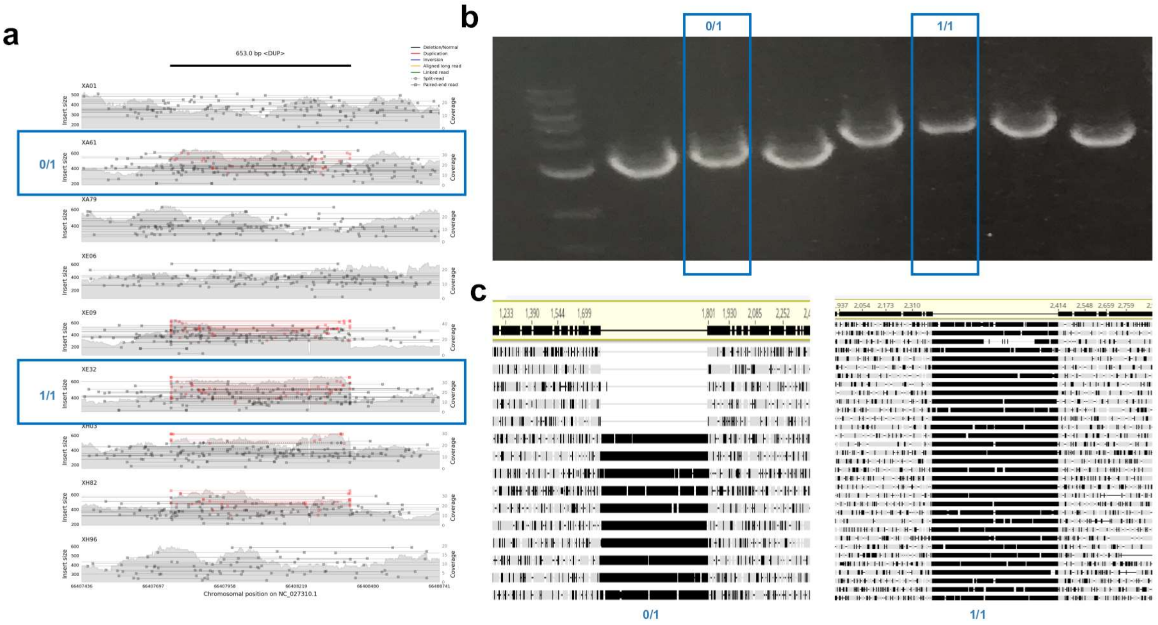

Supplementary Figure 13. Example of congruence between SV/genotype calls and data generated by MinION amplicon sequencing. **a** SV-plaudit duplication plot for 9 tested salmon individuals. Circled in blue are one 0/1 genotype call and one 1/1 genotype call. **b** Agarose gel image for the same individuals, with two calls highlighted in the gel image, showing the same genotypes. The DNA ladder in the left lane is a 1 kb DNA Ladder from New England Biolabs (Catalog# N3232S). **c** Alignments of amplicons sequenced for the same samples highlighted in **b** .

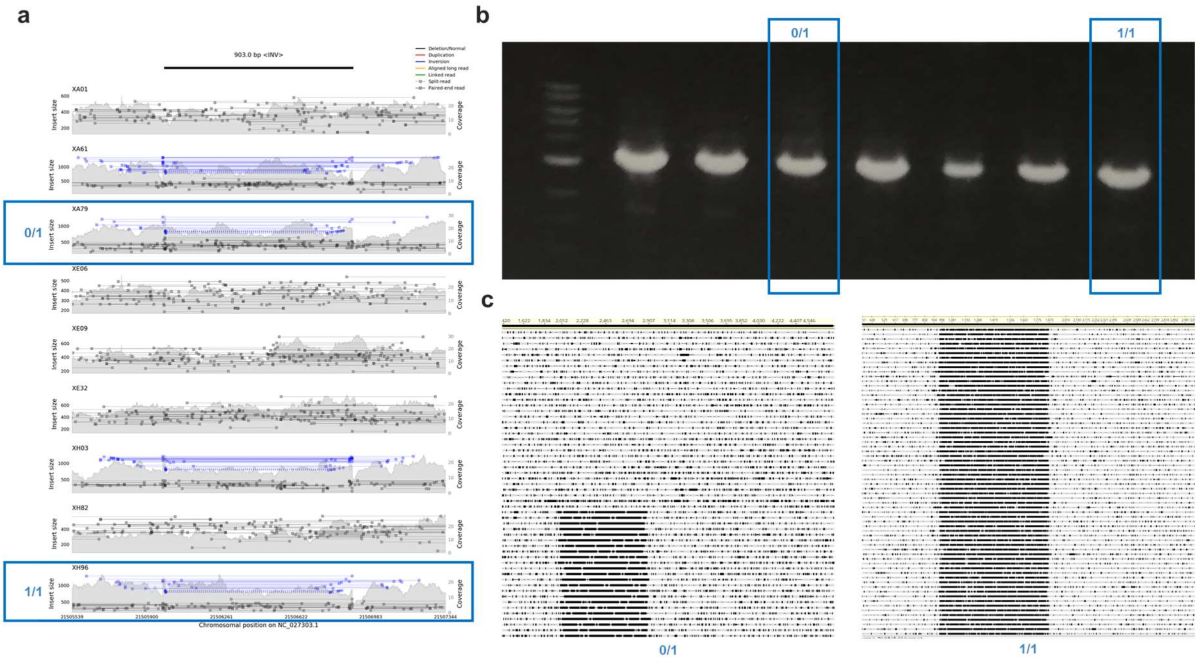

Supplementary Figure 14. Example of congruence between SV/genotype calls and data generated by MinION amplicon sequencing. **a** SV-plaudit inversion plot for 9 tested salmon individuals. Circled in blue are one 0/1 genotype call and one 1/1 genotype call. **b** Agarose gel image for the same individuals, with two calls highlighted in the gel image, showing the same genotypes. The DNA ladder in the left lane is a 1 kb DNA Ladder from New England Biolabs (Catalog# N3232S). **c** Alignments of amplicons sequenced for the same samples highlighted in **b**.

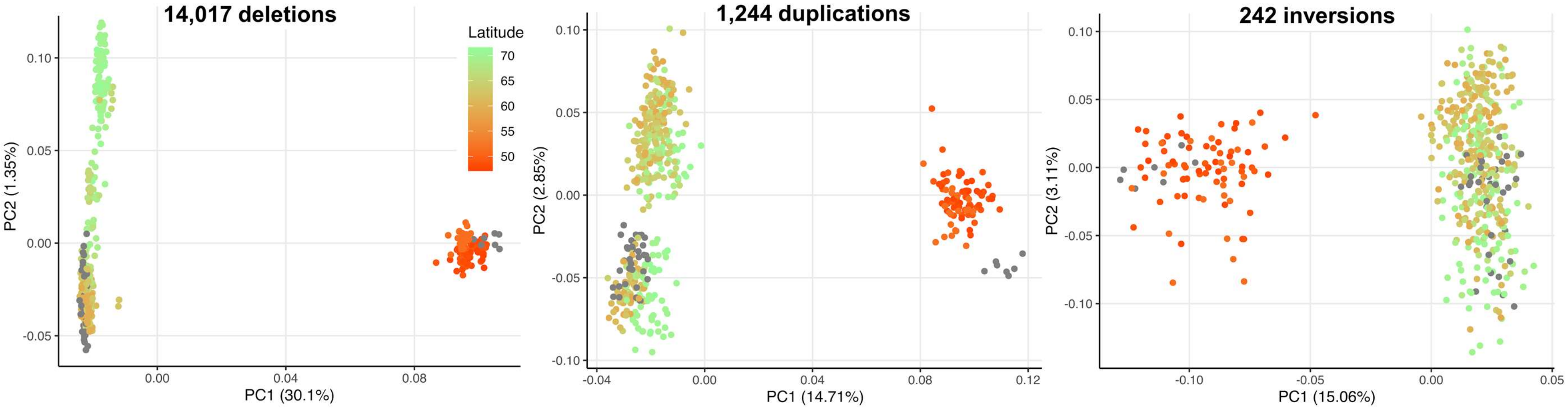

Supplementary Figure 15. PCAs showing the same data presented in Fig. 1g-i (main text), except visualized according to latitude. For all SV types, PC1 separates European and Canadian salmon (orange symbols; wild Canadian fish were all sampled from lower latitudes than all wild European salmon). For the deletion genotypes, PC2 separates populations according to latitude, which is expected (see main text). The grey symbols depict farmed salmon individuals.

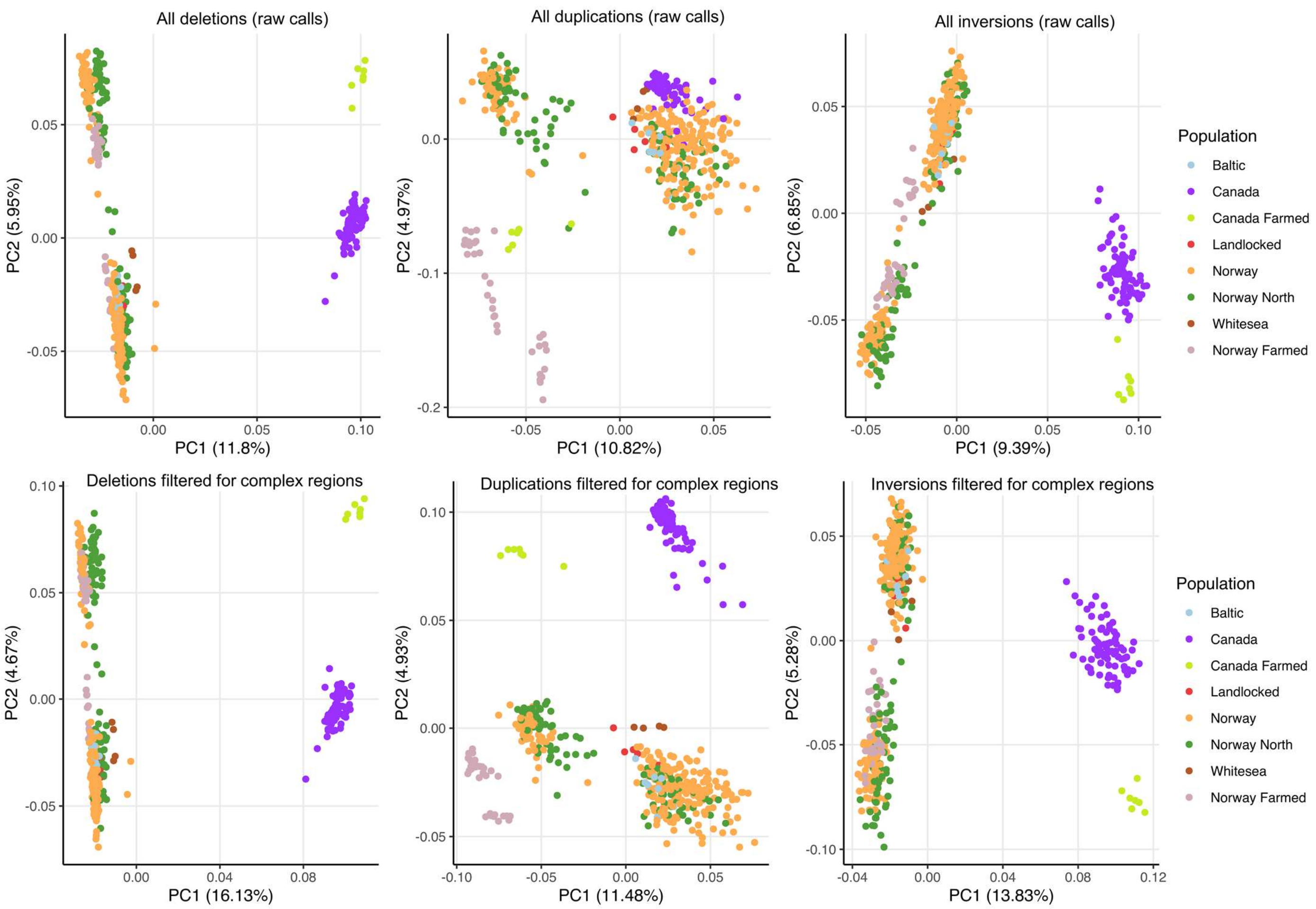

Supplementary Figure 16. PCA analyses done on SV genotype calls prior to SV-plaudit curation for i) the complete set of unfiltered SV genotype calls prior to filtering complex regions or SV-plaudit curation (top panel) and ii) the subset of SV genotype calls remaining after filtering complex regions in the genome, but prior to SV-plaudit curation (bottom panel). Consistent with the filtering of unreliable SV calls during SV-plaudit curation, both sets fail to capture expected population genetic structure at high resolution. For instance, in both datasets, duplications fail to separate Canadian and European salmon. Further, both datasets fail to split European salmon by latitude for all SV types (see Supplementary Figure 15) and erroneously place farmed salmon populations. For references supporting these deductions, see main text.

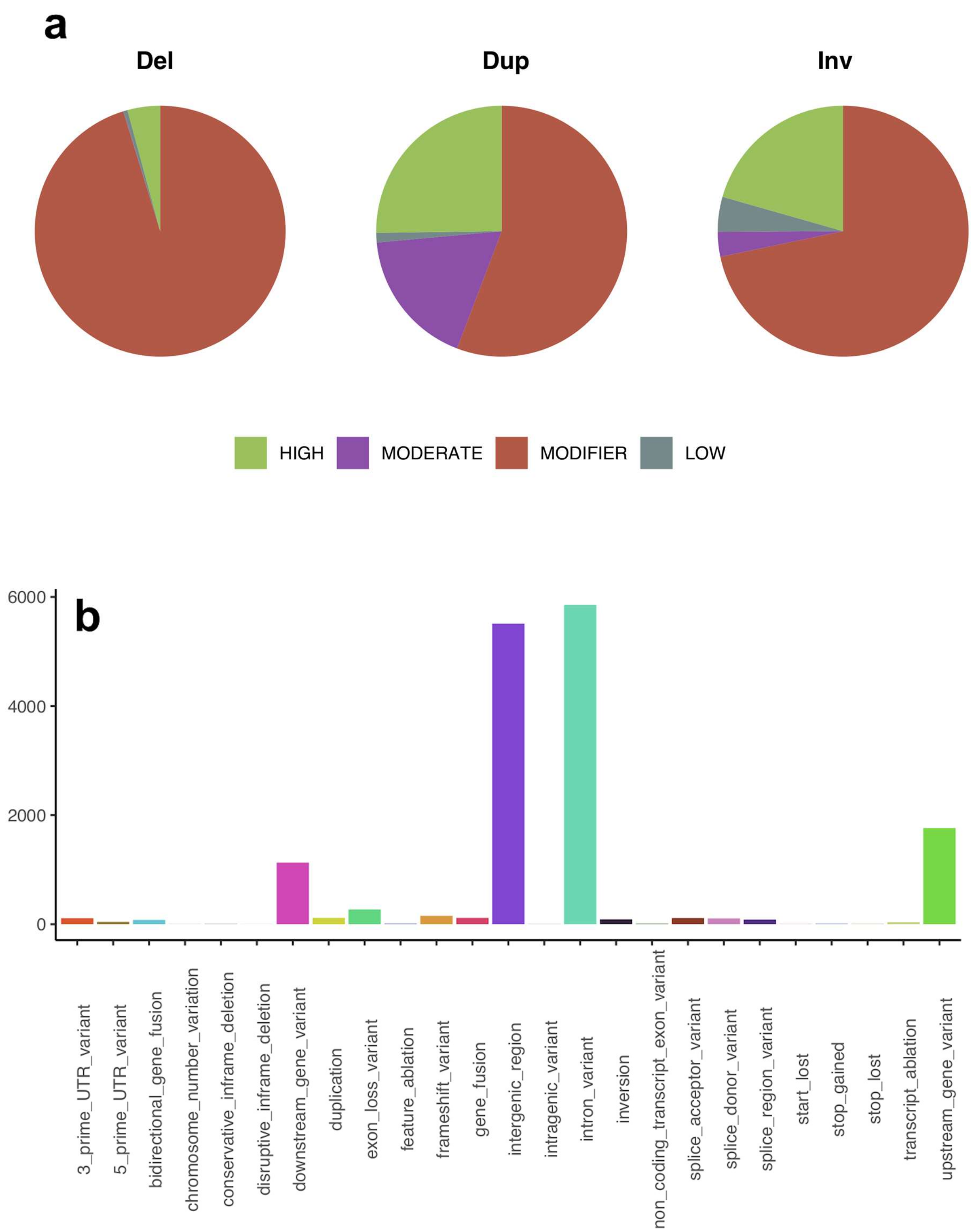

Supplementary Figure 17. SV annotation by SnpEff **a** Proportion of putative impacts predicted by snpEff for each SV type shown for all high-confidence Atlantic salmon SVs. **b** Number of SVs for each effect as annotated by snpEff.

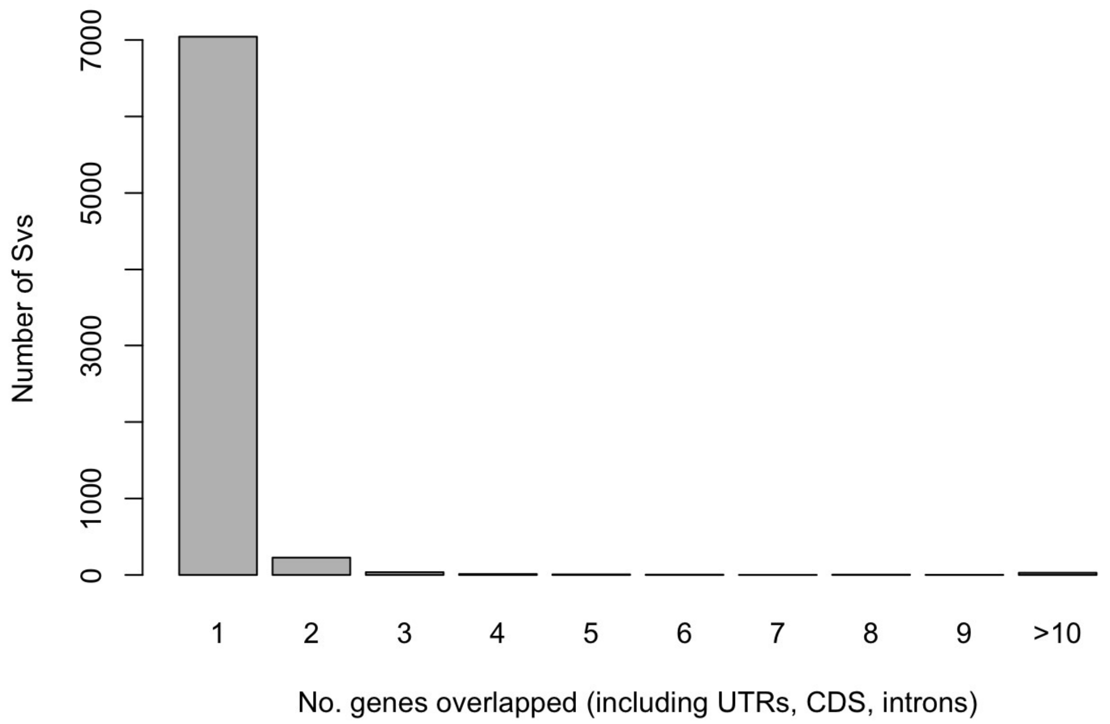

Supplementary Figure 18. Overlap between high confidence SVs and protein coding genes in the ICSASG_v2 annotation.

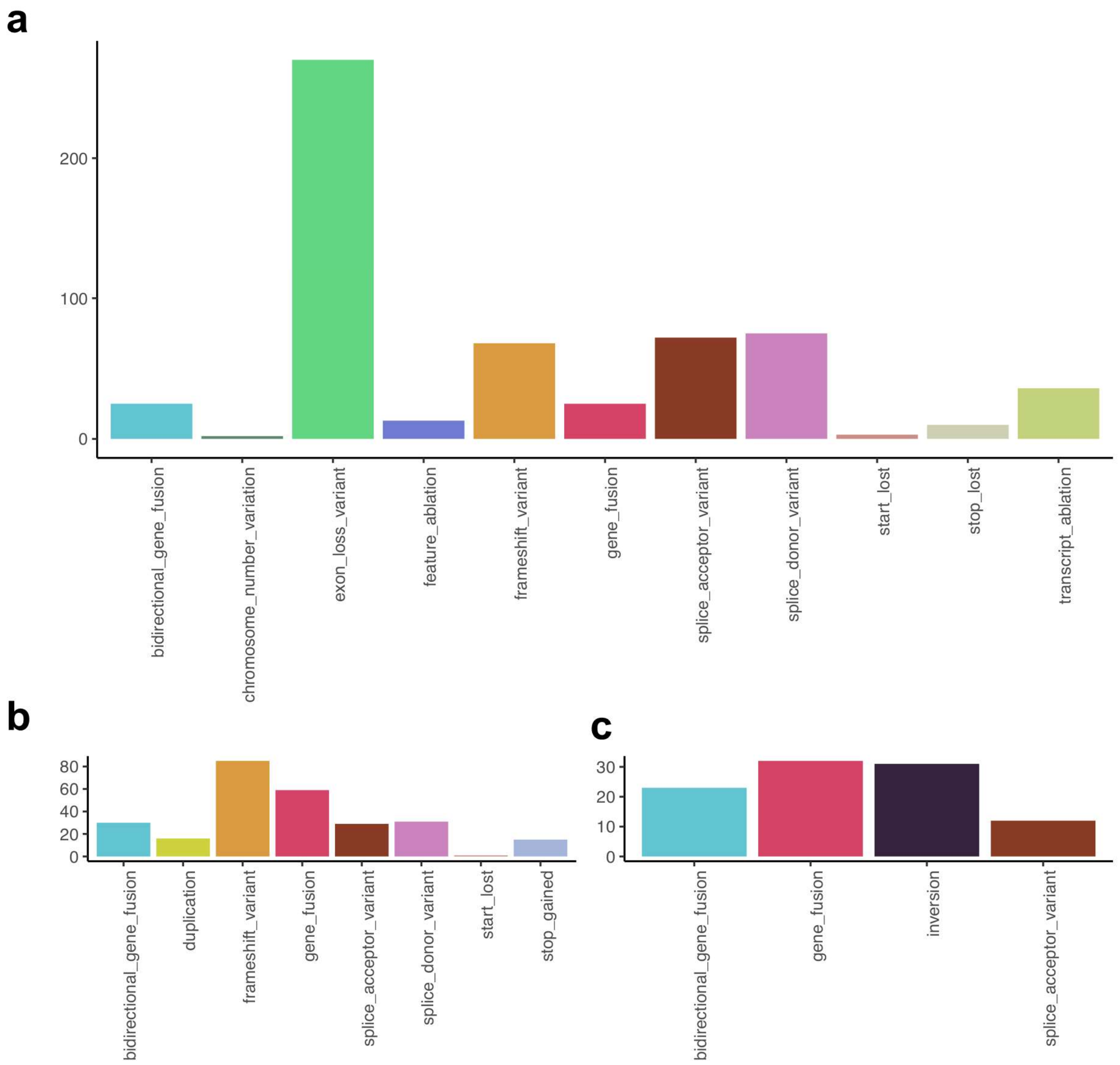

Supplementary Figure 19. Number of high impact annotations per snpEff effect for high-confidence Atlantic salmon SVs. **a** deletions, **b** duplications, **c** inversions.

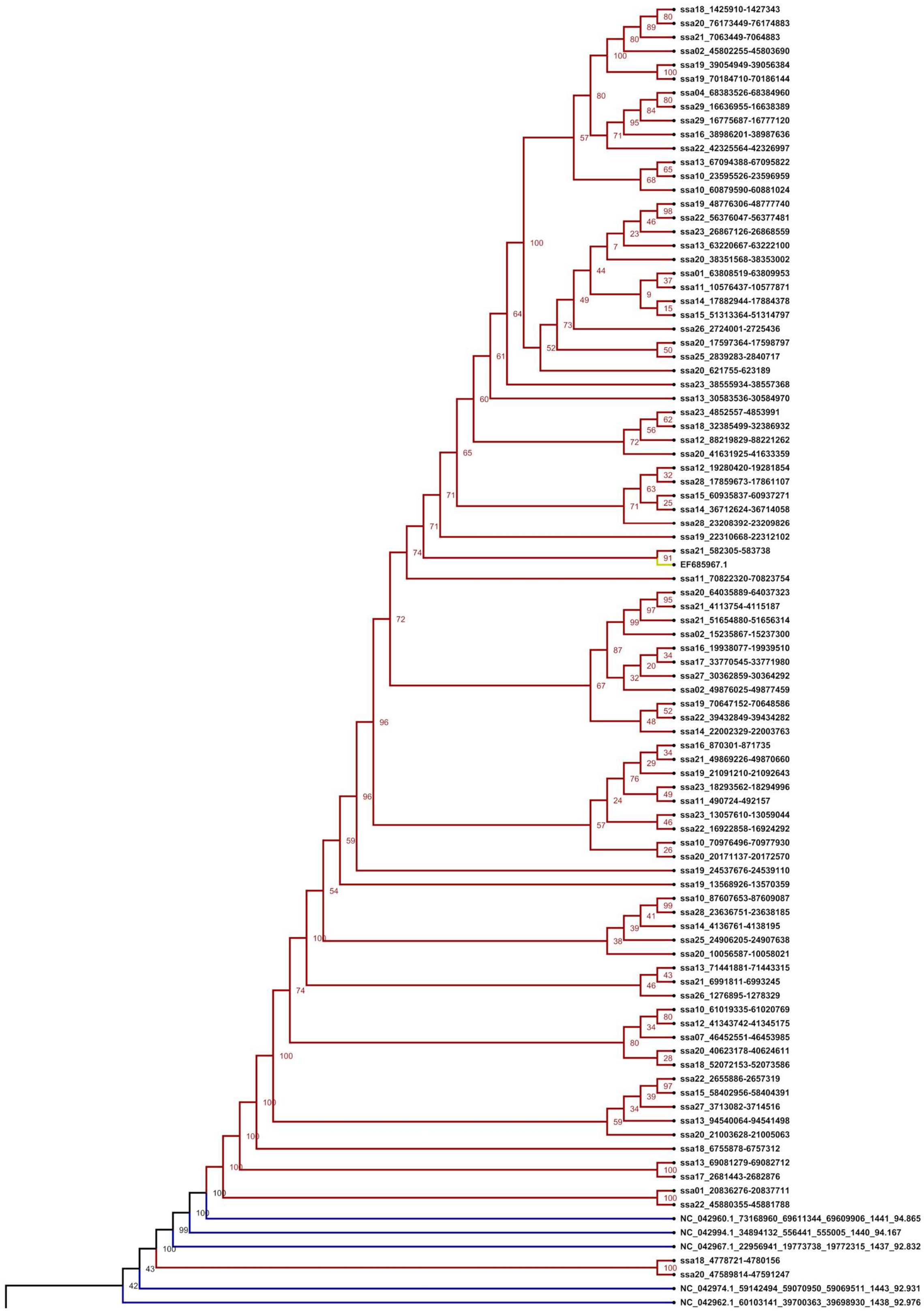

Cont. on next page

Cont. from last page

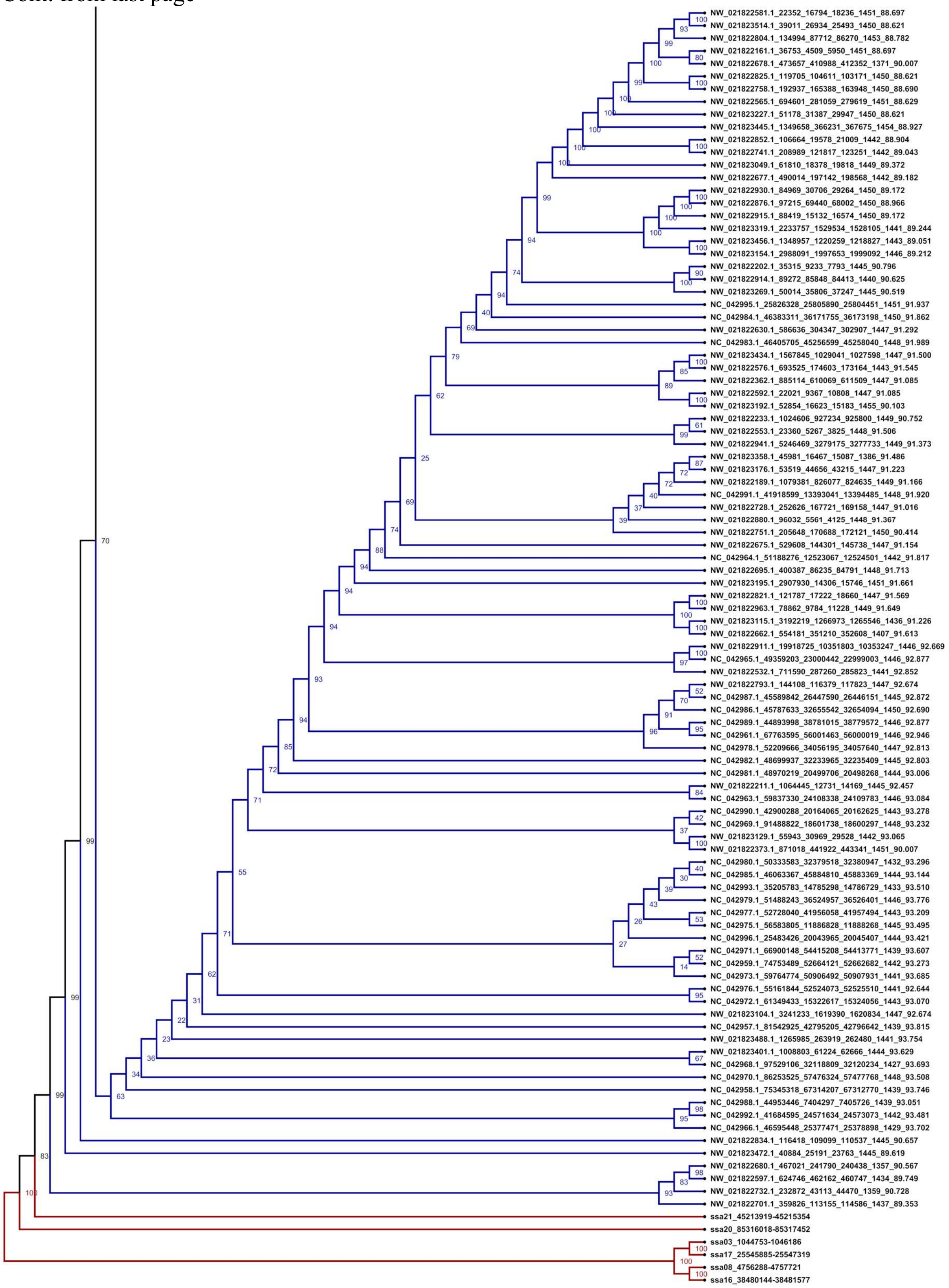

Supplementary Figure 20. Maximum likelihood tree presented in Main Text Fig. 2 including sample identifiers, genomic locations of pTSsa2 sequences and bootstrap values. Alignment provided in Supplementary Data 8.

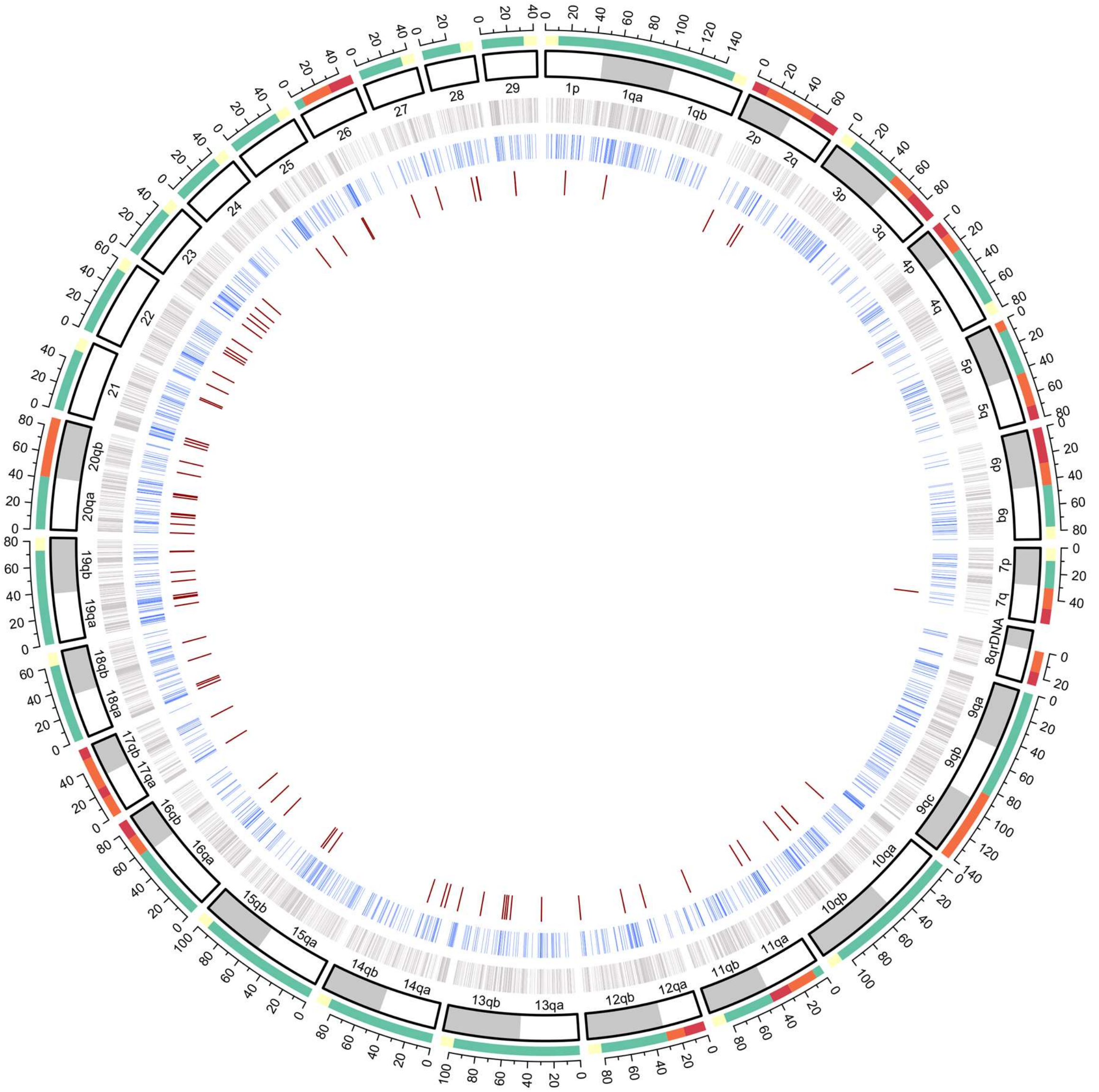

Supplementary Figure 21. Circos plot showing the genomic locations of pTSsa2 sequences in the Atlantic salmon genome for i) 94 polymorphic deletion sequences used in the tree shown in Fig. 2 (red lines, internal track), ii) 1,519 sequences within 1432-1436bp deletion peak before SV-plaudit filtering, sharing ≥95% and ≥ 98% coverage BLAST identity and coverage to the reference pTSsa2 sequence (blue lines, middle track) and iii) all pTSsa2 in the unmasked Atlantic salmon genome sharing ≥95% and ≥ 98% coverage BLAST identity to the reference pTSsa2 (grey line, outer track). The colours on the outside of chromosome arms, which are depicted with established *S. salar* nomenclature[17], depict the regions highlighted in Fig. 1b and Supplementary Fig. 2.

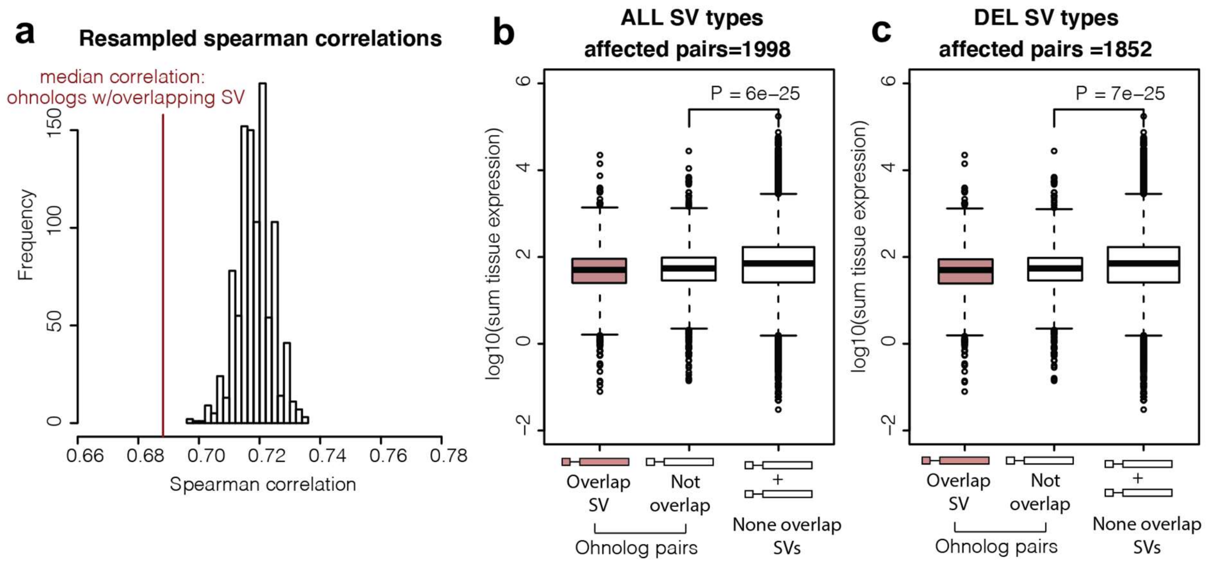

Supplementary Figure 22. Expression characteristics of ohnologs depending on SV overlap. **a** Spearman's correlation between Ss4R ohnolog pairs where one gene is overlapped by an SV in comparison to randomly resampled ohnolog pairs that do not overlap SVs. Also shown, both for all SVs (**b**) and just deletions (**c**), is the expression level of ohnologs (the sum of expression across 15 tissues), with different boxplots representing ohnologs overlapped by an SV (left) vs. the other gene in the same ohnolog pair (that does not overlap an SV) (middle) and ohnolog pairs where neither gene is overlapped by any SV. The *P* values shown on each of the plots were generated using paired Wilcoxon tests. Definition of box and whisker plots: the box spans the interquartile range, with the median (Q2) as a central bar, and respective upper and lower bounds representing the minimum and maximum values within the 25th percentile (Q1) and 75th percentile (Q3). The bounds of the upper and lower whisker are the largest and smallest values that lie within 1.5 times above Q3 and below Q1, respectively. Outliers out with these bounds are shown as individual points.

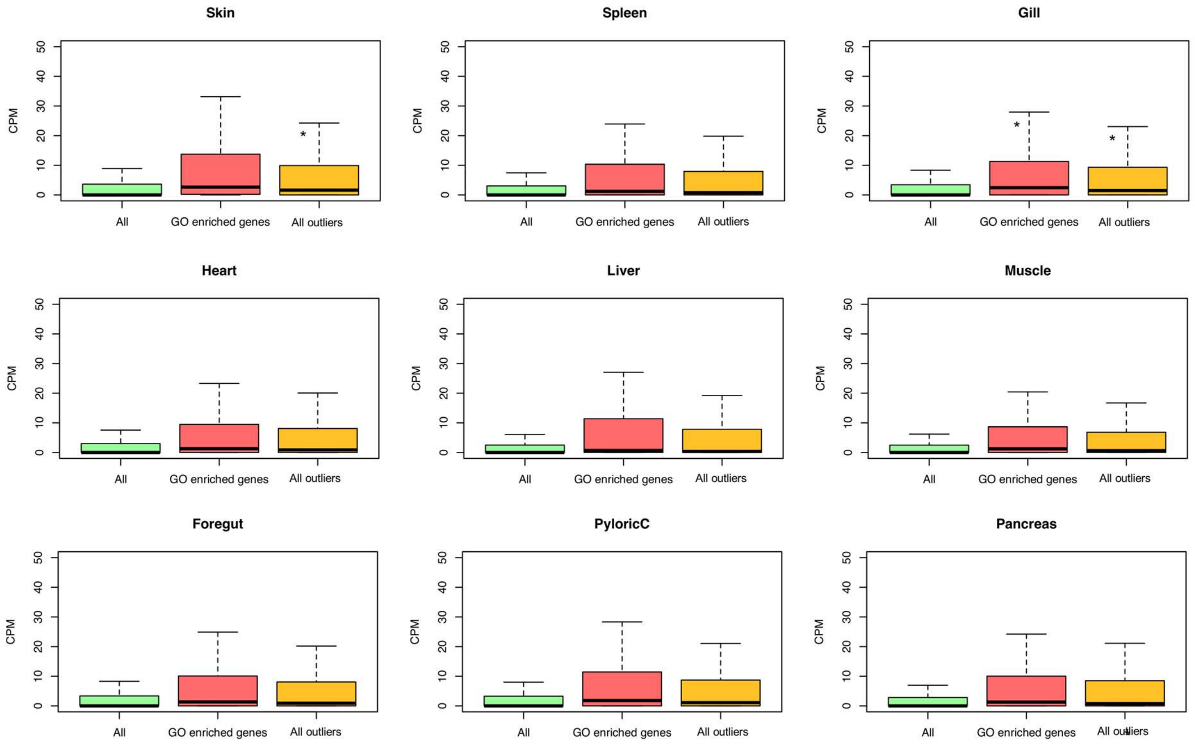

Supplementary Figure 23. Tissue expression levels (counts per million, CPM) comparing 44,469 genes in a transcriptome ('All') with genes linked by SnpEff to the 584 SV outliers ('All outliers') and the subset of 326 genes contributing to enriched biological processes by GO analysis ('GO enriched genes'). The asterisks shown indicate $P = 0.024$ (skin 'All outliers'), $P = 0.022$ (gill 'All outliers') and $P = 0.030$ (gill 'Go enriched genes'), which were drawn from two-sample t-tests done separately for each tissue comparing expression level (CPM) between a 44,469 global transcriptome set vs. i) the 584 gene subset and ii) the 326 gene subset. Results of two-sample t-tests for all tissues is shown for the 326 gene set in Supplementary Data 15. Definition of box and whisker plots: the box spans the interquartile range, with the median (Q2) as a central bar, and respective upper and lower bounds representing the minimum and maximum values within the 25th percentile (Q1) and 75th percentile (Q3). The bounds of the upper and lower whisker are the largest and smallest values that lie within 1.5 times above Q3 and below Q1, respectively.

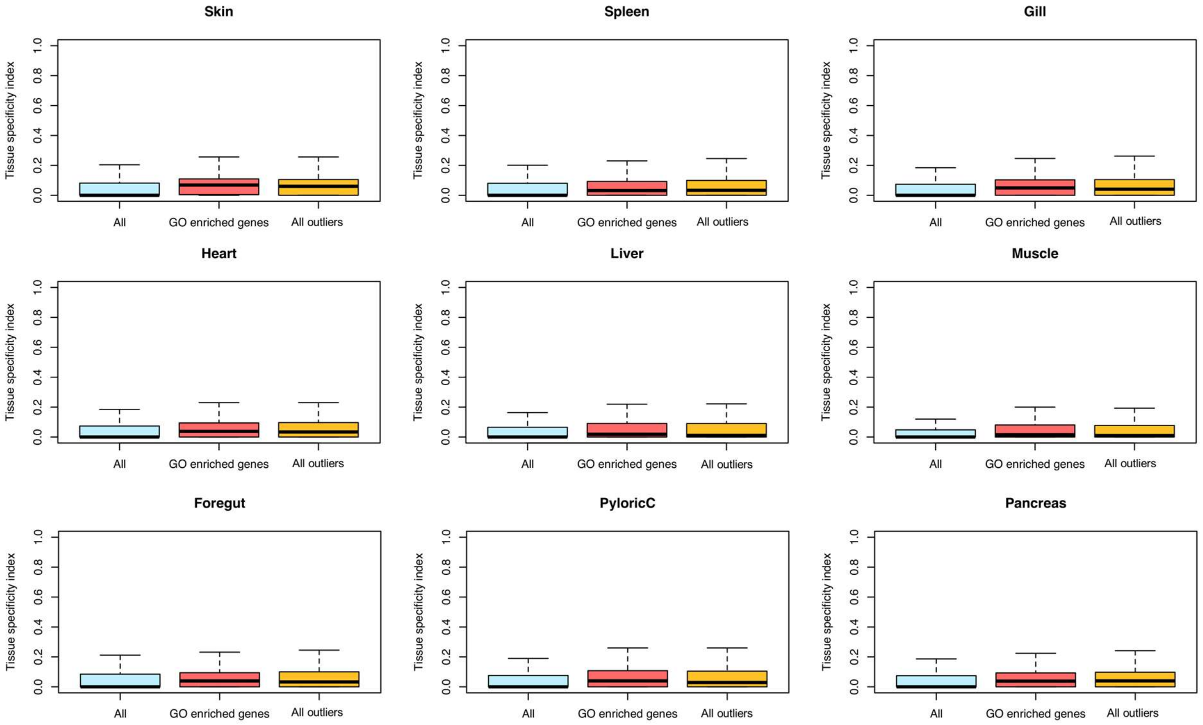

Supplementary Figure 24. Tissue specificity (see Methods) comparing 44,469 genes in a transcriptome ('All') with genes linked by SnpEff to the 584 SV outliers ('All outliers') and the subset of 326 genes contributing to enriched biological processes by GO analysis ('GO enriched genes'). Brain is shown in Fig. 4d (main text). Definition of box and whisker plots: the box spans the interquartile range, with the median (Q2) as a central bar, and respective upper and lower bounds representing the minimum and maximum values within the 25th percentile (Q1) and 75th percentile (Q3). The bounds of the upper and lower whisker are the largest and smallest values that lie within 1.5 times above Q3 and below Q1, respectively.

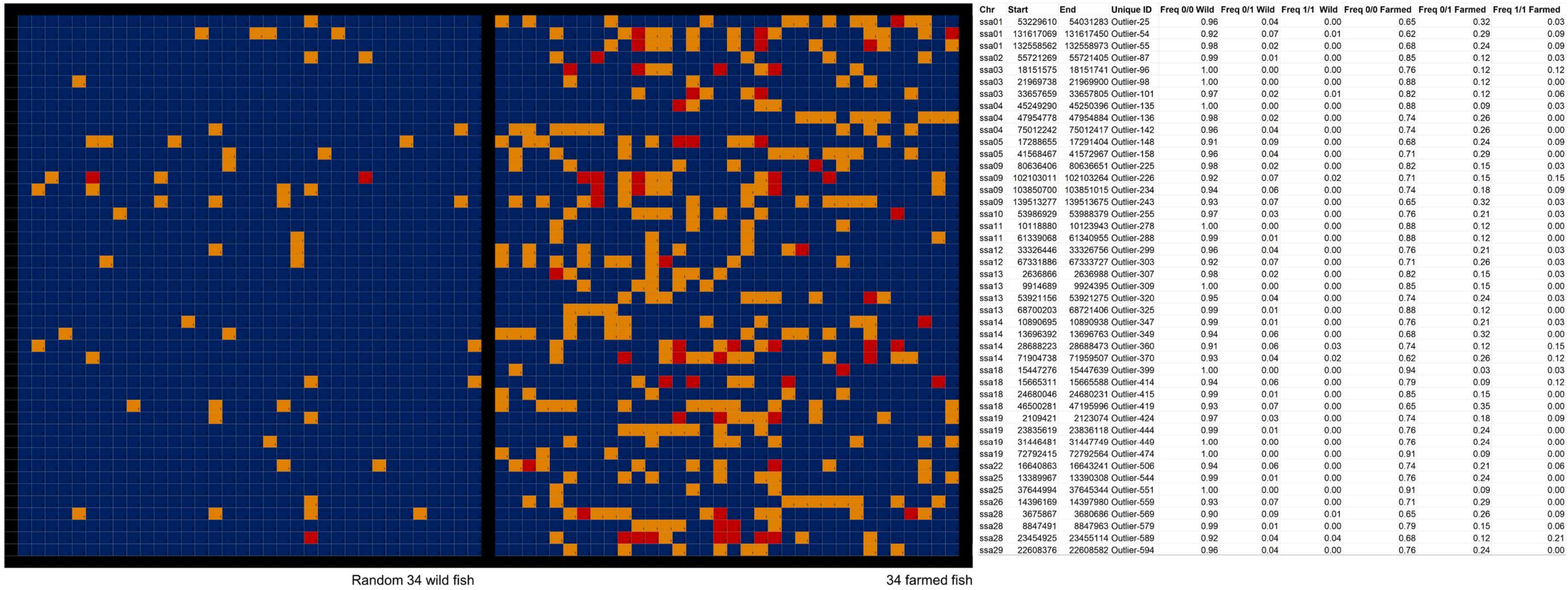

Chr	Start	End	Unique ID	Freq 0/0 Wild	Freq 0/1 Wild	Freq 1/1 Wild	Freq 0/0 Farmed	Freq 0/1 Farmed	Freq 1/1 Farmed
ssa01	53229610	54031283	Outlier-25	0.96	0.04	0.00	0.65	0.32	0.03
ssa01	131617069	131617450	Outlier-54	0.92	0.07	0.01	0.62	0.29	0.09
ssa01	132558562	132558973	Outlier-55	0.98	0.02	0.00	0.68	0.24	0.09
ssa02	55721269	55721405	Outlier-87	0.99	0.01	0.00	0.85	0.12	0.03
ssa03	18151575	18151741	Outlier-96	1.00	0.00	0.00	0.76	0.12	0.12
ssa03	21969738	21969900	Outlier-98	1.00	0.00	0.00	0.88	0.12	0.00
ssa03	33657659	33657805	Outlier-101	0.97	0.02	0.01	0.82	0.12	0.06
ssa04	45249290	45250396	Outlier-135	1.00	0.00	0.00	0.88	0.09	0.03
ssa04	47954778	47954884	Outlier-136	0.98	0.02	0.00	0.74	0.26	0.00
ssa04	75012242	75012417	Outlier-142	0.96	0.04	0.00	0.74	0.26	0.00
ssa05	17288655	17291404	Outlier-148	0.91	0.09	0.00	0.68	0.24	0.09
ssa05	41568467	41572967	Outlier-158	0.96	0.04	0.00	0.71	0.29	0.00
ssa09	80636406	80636651	Outlier-225	0.98	0.02	0.00	0.82	0.15	0.03
ssa09	102103011	102103264	Outlier-226	0.92	0.07	0.02	0.71	0.15	0.15
ssa09	103850700	103851015	Outlier-234	0.94	0.06	0.00	0.74	0.18	0.09
ssa09	139513277	139513675	Outlier-243	0.93	0.07	0.00	0.65	0.32	0.03
ssa10	53986929	53988379	Outlier-255	0.97	0.03	0.00	0.76	0.21	0.03
ssa11	10118880	10123943	Outlier-278	1.00	0.00	0.00	0.88	0.12	0.00
ssa11	61339068	61340955	Outlier-288	0.99	0.01	0.00	0.88	0.12	0.00
ssa12	33326446	33326756	Outlier-299	0.96	0.04	0.00	0.76	0.21	0.03
ssa12	67331886	67333727	Outlier-303	0.92	0.07	0.00	0.71	0.26	0.03
ssa13	2636866	2636988	Outlier-307	0.98	0.02	0.00	0.82	0.15	0.03
ssa13	9914689	9924395	Outlier-309	1.00	0.00	0.00	0.85	0.15	0.00
ssa13	53921156	53921275	Outlier-320	0.95	0.04	0.00	0.74	0.24	0.03
ssa13	68700203	68721406	Outlier-325	0.99	0.01	0.00	0.88	0.12	0.00
ssa14	10890695	10890938	Outlier-347	0.99	0.01	0.00	0.76	0.21	0.03
ssa14	13696392	13696763	Outlier-349	0.94	0.06	0.00	0.68	0.32	0.00
ssa14	28688223	28688473	Outlier-360	0.91	0.06	0.03	0.74	0.12	0.15
ssa14	71904738	71959507	Outlier-370	0.93	0.04	0.02	0.62	0.26	0.12
ssa18	15447276	15447639	Outlier-399	1.00	0.00	0.00	0.94	0.03	0.03
ssa18	15665311	15665588	Outlier-414	0.94	0.06	0.00	0.79	0.09	0.12
ssa18	24680046	24680231	Outlier-415	0.99	0.01	0.00	0.85	0.15	0.00
ssa18	46500281	47195996	Outlier-419	0.93	0.07	0.00	0.65	0.35	0.00
ssa19	2109421	2123074	Outlier-424	0.97	0.03	0.00	0.74	0.18	0.09
ssa19	23835619	23836118	Outlier-444	0.99	0.01	0.00	0.76	0.24	0.00
ssa19	31446481	31447749	Outlier-449	1.00	0.00	0.00	0.76	0.24	0.00
ssa19	72792415	72792564	Outlier-474	1.00	0.00	0.00	0.91	0.09	0.00
ssa22	16640863	16643241	Outlier-506	0.94	0.06	0.00	0.74	0.21	0.06
ssa25	13389967	13390308	Outlier-544	0.99	0.01	0.00	0.76	0.24	0.00
ssa25	37644994	37645344	Outlier-551	1.00	0.00	0.00	0.91	0.09	0.00
ssa26	14396169	14397980	Outlier-559	0.93	0.07	0.00	0.71	0.29	0.00
ssa28	3675867	3680686	Outlier-569	0.90	0.09	0.01	0.65	0.26	0.09
ssa28	8847491	8847963	Outlier-579	0.99	0.01	0.00	0.79	0.15	0.06
ssa28	23454925	23455114	Outlier-589	0.92	0.04	0.04	0.68	0.12	0.21
ssa29	22608376	22608582	Outlier-594	0.96	0.04	0.00	0.76	0.24	0.00

Supplementary Figure 25. Heatmap showing individual SV genotypes for 45 SV outliers linked to synapse genes in the salmon genome. SVs have been filtered to only include rare SVs in wild fish. The blue, yellow and red squares depict homozygous 0/0 (lacking SV), heterozygous 0/1 (one SV copy), and homozygous 1/1 (two SV copies) genotypes, respectively. Each column represents a different fish individual. The SVs are organized from top to bottom with respect to their location in the genome.

Supplementary Note 1. Python script used to extract gap regions in the ICSASG_v2 genome and convert the output to a BED file.

Obtained from: https://www.danielecook.com/generate-a-bedfile-of-masked-ranges-a-fasta-file/

Usage:

```
python generate_masked_ranges.py <fasta_file> > output_ranges.txt
```

Code:

```
#!bin/python

import gzip
import io
import sys
import os

# This file will generate a bedfile of the masked regions a fasta file.

# STDIN or arguments
if len(sys.argv) > 1:

    # Check file type
    if sys.argv[1].endswith(".fa.gz"):
        input_fasta = io.TextIOWrapper(io.BufferedReader(gzip.open(sys.argv[1])))
    elif sys.argv[1].endswith(".fa") or sys.argv[1].endswith(".txt"):
        input_fasta = file(sys.argv[1],'r')
    else:
        raise Exception("Unsupported File Type")
else:
    print """
    \tUsage:\n\t\tgenerate_masked_ranges.py <fasta file | .fa or .fa.gz> <chrome find> <chrome
replace>

    \t\t'Chrome find' and 'chrome replace' are used to find and replace the name of a chromsome. For
example,
    \t\treplacing CHROMSOME_I with chr1 can be accomplished by using the command as follows:
    \t\t\tpython generate_masked_ranges.py my_fasta.fa CHROMSOME_ chr
    \t\tOutput is to stdout
    """
    raise SystemExit


n, state = 0, 0 # line, character, state (0=Out of gap; 1=In Gap)
chrom, start, end = None, None, None

with input_fasta as f:
    for line in f:
        line = line.replace("\n","")
        if line.startswith(">"):
            # Print end range
            if state == 1:
```

```
            print '\t'.join([chrom ,str(start), str(n)])
            start, end, state  = 0, 0, 0
        n = 0 # Reset character
        chrom = line.split(" ")[0].replace(">","")
        # If user specifies, replace chromosome as well
        if len(sys.argv) > 2:
            chrom = chrom.replace(sys.argv[2],sys.argv[3])
    else:
        for char in line:
            if state == 0 and char == "N":
                state = 1
                start = n
            elif state == 1 and char != "N":
                state = 0
                end = n
                print '\t'.join([chrom ,str(start), str(end)])
            else:
                pass

            n += 1 # First base is 0 in bed format.

# Print mask close if on the last chromosome.
if state == 1:
        print '\t'.join([chrom ,str(start), str(n)])
        start, end, state  = 0, 0, 0
```

Supplementary Note 2. Snakefiles and associated code for SV calling pipeline

Snakefile1 main rules:

SAMPLES = ["Sample1", "Sample2"]

snakefiles = "snakefiles/"

include: snakefiles + "align"

include: snakefiles + "smoove"

rule all:

input:

"smoove/results/genotyped/paste/cohort.smoove.square.anno.vcf.gz",

"indexcov/index.html"

Snakefile 1:

rule bwa_index_reference:
input:

"/users/r01acb15/sharedscratch/reference/ICSASG_v2.fa"

output:

"/users/r01acb15/sharedscratch/reference/ICSASG_v2.fa"

log:

"bwa_index_reference.log"

shell:

"bwa index {input} &> {log}"

rule bwa_map:

input:

reference = "/users/r01acb15/sharedscratch/reference/ICSASG_v2.fa",

forward = "data/samples/{sample}_1.fastq",

reverse = "data/samples/{sample}_2.fastq"

output:

"mapped_reads/{sample}.bam"

params:

```
rg="@RG\\tID:{sample}\\tSM:{sample}\\tLB:lib1"

log:

"bwa_{sample}.log"

threads:

16

shell:

"(bwa mem -R '{params.rg}' -t {threads} {input.reference}

{input.forward} {input.reverse} | "

"samtools view -Sb - "

"> {output}) "

"2> {log}"

rule samtools_sort:

input:

"mapped_reads/{sample}.bam"

output:

"sorted_reads/{sample}.bam"

log:

"sort_{sample}.log"

shell:

"samtools sort -T sorted_reads/{wildcards.sample} "
"-O bam {input} > {output} "

"2> {log} "

rule samtools_index:

input:

"sorted_reads/{sample}.bam"

output:

"sorted_reads/{sample}.bam.bai"
```

```
shell:
"samtools index {input}"

rule goleft_indexcov:

input:

bam=expand("sorted_reads/{sample}.bam", sample=SAMPLES),

bai=expand("sorted_reads/{sample}.bam.bai", sample=SAMPLES)

output:

"indexcov/index.html"

log:

"indexcov.log"

shell:

"goleft indexcov -d indexcov/ {input.bam} 2> {log}"
```

Snakefile 2:

```
REF = "/users/r01acb15/sharedscratch/reference/ICSASG_v2.fa",
EXCLUDE =

"/users/r01acb15/sharedscratch/reference/high_depth_regions.min100_cluster.Nga
ps.bed",

GFF = "/users/r01acb15/sharedscratch/reference/Salmo_salar-annotation.gff3"
rule call:

input:

bam="sorted_reads/{sample}.bam"

output:

"smoove/results/{sample}-smoove.genotyped.vcf.gz"

conda:

"envs/smoove2.3.yaml"

shell:

"smoove call --outdir smoove/results/ --exclude {EXCLUDE} –name

{wildcards.sample} --fasta {REF} -p 1 --genotype {input.bam}"

rule merge:

input:
```

```
vcf=expand("smoove/results/{sample}-smoove.genotyped.vcf.gz",
sample=SAMPLES)

output:

"smoove/results/merged.sites.vcf.gz"

conda:

"envs/smoove2.3.yaml"

shell:

"smoove merge --name merged -f {REF} --outdir ./smoove/results/

{input.vcf}"

rule genotype:

input:

merge="smoove/results/merged.sites.vcf.gz",

bam="sorted_reads/{sample}.bam"

output:

"smoove/results/genotyped/{sample}-joint-smoove.genotyped.vcf.gz"

conda:

"envs/smoove2.3.yaml"

shell:

"smoove genotype -d -x -p 1 --name {wildcards.sample}-joint –outdir
smoove/results/genotyped/ --fasta {REF} --vcf {input.merge} {input.bam}"

rule paste:

input:

vcf=expand("smoove/results/genotyped/{sample}-jointsmoove.

genotyped.vcf.gz", sample=SAMPLES)

output:

"smoove/results/genotyped/paste/cohort.vcf.gz"

conda:

"envs/smoove2.3.yaml"
```

shell:
"smoove paste --name cohort {input.vcf}"

rule annotate:

input:

"smoove/results/genotyped/paste/cohort.vcf.gz"

output:

"smoove/results/genotyped/paste/cohort.smoove.square.anno.vcf.gz"

conda:

"envs/smoove2.3.yaml"

shell:

"smoove annotate --gff {GFF} {input} | bgzip -c > {output}"

Conda environment to run smoove step of Snakefile:

This environment should be inserted into a .yaml file and executre with conda.

name: smoove2.3

channels:

- bioconda
- conda-forge
- defaults

dependencies:

- anaconda=custom=py27h4a00acb_0
- asn1crypto=0.24.0=py27_3
- backports=1.0=py_2
- backports.functools_lru_cache=1.5=py_1
- backports_abc=0.5=py_1
- bcftools=1.9=h4da6232_0
- bedtools=2.27.1=he941832_2
- blas=1.0=mkl
- boto3=1.9.4=py_0
- botocore=1.12.4=py_0
- bzip2=1.0.6=h470a237_2
- ca-certificates=2019.6.16=hecc5488_0
- certifi=2019.6.16=py27_0
- cffi=1.11.5=py27h5e8e0c9_1
- cryptography=2.3.1=py27hdffb7b8_0
- cryptography-vectors=2.3.1=py27_0
- curl=7.61.0=h93b3f91_2
- cycler=0.10.0=py_1
- cytoolz=0.9.0.1=py27h470a237_1

- dbus=1.13.0=h3a4f0e9_0
- docutils=0.14=py27_1
- duphold=0.0.9=0
- enum34=1.1.6=py27_1
- expat=2.2.5=hfc679d8_2
- fontconfig=2.13.1=h65d0f4c_0
- freetype=2.9.1=h6debe1e_4
- functools32=3.2.3.2=py27_2
- futures=3.2.0=py27_0
- gawk=4.2.1=h470a237_0
- gettext=0.19.8.1=h5e8e0c9_1
- glib=2.55.0=h464dc38_2
- gsort=0.0.6=1
- gst-plugins-base=1.12.5=hde13a9d_0
- gstreamer=1.12.5=h61a6719_0
- htslib=1.9=hc238db4_4
- icu=58.2=hfc679d8_0
- idna=2.7=py27_2
- intel-openmp=2019.0=117
- ipaddress=1.0.22=py_1

Supplementary Note 3: Custom R script used to obtain Fst values from random comparisons and establish probability value for outlier SVs

```
#######200 iterations to obtain Fst values from random comparisons######

###"Indivduals.txt" is a file with the IDs of the animals in the farmed and South Norway populations. One ID per line.

###"TOTAL2mb_highqual.vcf" is VCF file with the calls for the high quality SVs

#We calculate Fst values for 200 random subsets of 34 and 257 animals

for i in {1..200}
do
  shuf -n 34 individuals.txt > small_pop.txt

##Obtain 34 random animals
  grep -w -v -F -f small_pop.txt individuals.txt > large_pop.txt

##Select the remaining 257 animals
  vcftools --vcf TOTAL2mb_highqual.vcf --weir-fst-pop small_pop.txt --weir-fst-pop large_pop.txt --out fst_pop1_vs_pop2                ##Perform Fst calculation using VCFtools
  cat fst_pop1_vs_pop2.weir.fst >> fst_results_n200.txt

##Save the results of all the iterations to the file "fst_results_n200.txt"
done


#########P-values for each individual high quality SV########

###"Farmed-vs-SouthNorwayWild.fst" is a file with the VCFtools Fst calculation output for the farmed vs wild South Norway comparison for the high-quality SVs. One SV per line, first and second columns have the position of the SNP, the third column has the Fst value.
cat Farmed-vs-SouthNorwayWild.fst | while read line

##We process one SV each time

do

FST=$(echo $line | awk '{print $3;}')

#We extract the Fst value for the current SV

echo $line | awk '{print $1,$2;}' | sed 's/ /\t/g' - | grep -w -f - fst_results_n200.txt | cut -f3 - > temporal.txt

#From the previous 200 random Fst iterations, we select the lines for the current SV

OVER=$(awk -v var="$FST" '$1>var{c++} END{print c+0}' temporal.txt)

#We obtain the number of random iterations that resulted in a higher Fst than the one obtain in random vs wild South Norway for the current SNP

echo $OVER >> number_below_fst.txt
```

#We save the number of higher random Fst in a file, the number for each SV in one line
PVAL=$(bc <<< "scale=4;$OVER/200")

#We calculate the proportion of higher random Fst by dividing the number by 200. This is the p-value
echo

$PVAL >> p_value.txt

#We store the p-values in a file, one line per SV
done

paste Farmed-vs-SouthNorwayWild.fst number_below_fst.txt p_value.txt > Farmed-vs-SouthNorwayWild-with-pval.fst

#We merge the output of VCFtools Fst calculation with the p-values.
