# Supplementary material for: The structural variation landscape in 492 Atlantic salmon genomes
Source: Nat Commun. 2020 Oct 14;11:5176. doi: 10.1038/s41467-020-18972-x (PMC7560756; doi:10.1038/s41467-020-18972-x)
Supplement: Supplementary file 2 — Reporting Summary [file 41467_2020_18972_MOESM2_ESM.pdf]

nature research

Corresponding author(s): Daniel J. Macqueen; Sigbjørn Lien

Last updated by author(s): Sep 16, 2020

Reporting Summary

Nature Research wishes to improve the reproducibility of the work that we publish. This form provides structure for consistency and transparency in reporting. For further information on Nature Research policies, see our Editorial Policies and the Editorial Policy Checklist.

Statistics

For all statistical analyses, confirm that the following items are present in the figure legend, table legend, main text, or Methods section.

n/a	Confirmed	
☐	☒	The exact sample size (*n*) for each experimental group/condition, given as a discrete number and unit of measurement
☐	☒	A statement on whether measurements were taken from distinct samples or whether the same sample was measured repeatedly
☐	☒	The statistical test(s) used AND whether they are one- or two-sided<br>*Only common tests should be described solely by name; describe more complex techniques in the Methods section.*
☐	☒	A description of all covariates tested
☐	☒	A description of any assumptions or corrections, such as tests of normality and adjustment for multiple comparisons
☐	☒	A full description of the statistical parameters including central tendency (e.g. means) or other basic estimates (e.g. regression coefficient) AND variation (e.g. standard deviation) or associated estimates of uncertainty (e.g. confidence intervals)
☐	☒	For null hypothesis testing, the test statistic (e.g. *F*, *t*, *r*) with confidence intervals, effect sizes, degrees of freedom and *P* value noted<br>*Give P values as exact values whenever suitable.*
☒	☐	For Bayesian analysis, information on the choice of priors and Markov chain Monte Carlo settings
☒	☐	For hierarchical and complex designs, identification of the appropriate level for tests and full reporting of outcomes
☒	☐	Estimates of effect sizes (e.g. Cohen's *d*, Pearson's *r*), indicating how they were calculated

Our web collection on statistics for biologists contains articles on many of the points above.

Software and code

Policy information about availability of computer code

Data collection	Bioinformatic data was collected and analysed using open source or freely available software (mainly Unix, R or Python) specifically: BWA v0.7.13, Samtools v0.1.19, samplot v1.01, Indexcov goleft v0.2.1, Bedtools v2.27, mosdepth v0.2.3, Smoove v2.3, SVtyper v0.7.0, SV-plaudit (no version variations), cyvcf2 v0.11.5, SnpEff v4.3, TopGo (v2.26.0), Mafft v7.0, IQTree v1.6.12, Guppy v2.3.7, Orthofinder v2.4.0, Macse v2.03, TreeBeST v1.9.2, iadhore v3.0, Genrich v.06 , VCFtools v0.1.1, Snakemake v.3.11.0, rstatix v0.6.0, GGplot2 v3.3.2, NGSadmix v32 and BLAST (done using the NCBI Web BLAST server: https://blast.ncbi.nlm.nih.gov/Blast.cgi).<br><br>All custom code is reported: Python script used to identify regions in ICSASG_v2 genome and convert output to BED file: Supplementary Note 1. Snakefile and associated code for SV detection pipeline: Supplementary Note 2. R script used to obtain FST values from random comparisons and establish probability value for outlier SVs: Supplementary Note 3. Code to define orthogroups and build gene trees: https://gitlab.com/sandve-lab/salmonid_synteny (a zip file for the Gitlab repository is provided as Supplementary Data 7). Code to identify Atlantic salmon ohnolog pairs from ortholog groups and gene trees: https://gitlab.com/sandve-lab/defining_duplicates (a zip file for the Gitlab repository is provided as Supplementary Data 8). Code to analyse overlaps between SVs, ohnologs and ATAC-Seq data: https://gitlab.com/ssandve/atlantic_salmon_sv_ohnolog_analyses (a zip file for the Gitlab repository is provided as Supplementary Data 9).<br><br>Commercial programmes used: Geneious Prime 2019.1.1
Data analysis	As above - data collection and analyses described together, as often closely related for this study.

For manuscripts utilizing custom algorithms or software that are central to the research but not yet described in published literature, software must be made available to editors and reviewers. We strongly encourage code deposition in a community repository (e.g. GitHub). See the Nature Research guidelines for submitting code & software for further information.

Data

Policy information about availability of data

All manuscripts must include a data availability statement. This statement should provide the following information, where applicable:

- Accession codes, unique identifiers, or web links for publicly available datasets
- A list of figures that have associated raw data
- A description of any restrictions on data availability

The authors declare that all data supporting the findings of this study are available within the paper and its supplementary information files. Novel raw sequence data that support the findings of this study were deposited in the European Nucleotide Archive (ENA) or NCBI with the project accession PRJEB38061 [https://www.ebi.ac.uk/ena/browser/view/PRJEB38061] (genome re-sequencing data for 463 Atlantic salmon individuals), XXXXXX [Link] (MinION sequencing data) and PRJNA378201 [https://www.ncbi.nlm.nih.gov/bioproject/PRJNA378201/] (genome re-sequencing data for 9 Atlantic salmon individuals), and in ArrayExpress with the accession E-MTAB-9001 [https://www.ebi.ac.uk/arrayexpress/experiments/E-MTAB-9001/] (ATAC-Seq data). Individual sample accession numbers (ENA/NCBI) for all raw genome re-sequencing data (i.e. 492 Atlantic salmon genomes): ERS4601683-ERS4601685, ERS4601687-ERS4601688, ERS4601690-ERS4601696, ERS4601698-ERS4601700, ERS4601702-ERS4601710, ERS4601714-ERS4601719, ERS4601721, ERS4601723-ERS4601727, ERS4601732-ERS4601733, ERS4601735-ERS4601741, ERS4601743, ERS4601745-ERS4601748, ERS4601750-ERS4601754, ERS4601756-ERS4601760, ERS4601762, ERS4601764-ERS4601772, ERS4601774-ERS4601781, ERS4601783-ERS4601787, ERS4601789-ERS4601794, ERS4601796-ERS4601807, ERS4601809-ERS4601820, ERS4601822-ERS4601830, ERS4601832-ERS4601837, ERS4601839, ERS4601842-ERS4601850, ERS4601854-ERS4601855, ERS4601857-ERS4601858, ERS4601860, ERS4601862, ERS4601865-ERS4601867, ERS4601869, ERS4601871, ERS4601873-ERS4601876, ERS4601878-ERS4601880, ERS4601882-ERS4601885, ERS4601887-ERS4601904, ERS4601906-ERS4601907, ERS4601910-ERS4601911, ERS4601913-ERS4601931, ERS4601933-ERS4601936, ERS4601938-ERS4601939, ERS4601941-ERS4601946, ERS4601948-ERS4601955, ERS4601957-ERS4601961, ERS4601964, ERS4601966-ERS4601969, ERS4601971-ERS4601981, ERS4601983, ERS4601985-ERS4601986, ERS4601989-ERS4601996, ERS4601998-ERS4602011, ERS4602013, ERS4602015-ERS4602021, ERS4602023-ERS4602026, ERS4602028-ERS4602032, ERS4602034-ERS4602035, ERS4602037-ERS4602041, ERS4602043-ERS4602052, ERS4602054, ERS4602056-ERS4602067, ERS4602069, ERS4602071-ERS4602073, ERS4602075-ERS4602094, ERS4602097-ERS4602101, ERS4778562, ERS4778565-ERS4778566, ERS4778569, ERS4778572; SRR2070512, SRR2070597-SRR2070615, and SRX2843766-SRX2843774.

Field-specific reporting

Please select the one below that is the best fit for your research. If you are not sure, read the appropriate sections before making your selection.

☐ Life sciences　☐ Behavioural & social sciences　☒ Ecological, evolutionary & environmental sciences

For a reference copy of the document with all sections, see nature.com/documents/nr-reporting-summary-flat.pdf

Ecological, evolutionary & environmental sciences study design

All studies must disclose on these points even when the disclosure is negative.

Study description

Structural variation analysis of 492 Atlantic salmon individuals using short-read whole genome sequencing

Research sample

Paired-end whole genome sequencing data (mean 8.1x coverage, 2 x 100-150 bp) for 492 Atlantic salmon on several different platforms (Supplementary Table 1 in paper). We sampled n=80 wild Canadian individuals from 8 sites, n=359 Norwegian individuals from 52 sites (including n=5 landlocked dwarf salmon), n=8 Baltic individuals from a single site and n=4 White sea individuals from a single site. Whole genome sequencing data was generated for 21 farmed salmon individuals (n=12 individuals from Mowi ASA; n=9 samples from Xelect Ltd) and downloaded for a further 20 individuals. Individual sample accession numbers are given in Supplementary Table 1 and the Data Availability section.

The rationale for the choice of these samples was i) to provide a representation of multiple individuals from within the major Atlantic salmon phylogeographic groups including both wild and farmed populations and ii) to provide sufficient paired end sequencing coverage per sample to allow for reliable structural variation calls to be generated. These two aspects of the research sample were essential to achieve the study objectives of understanding the distribution and role of SVs in the Atlantic salmon genome.

Sampling strategy

The strategy for sampling in this study was not based on any statistical method to predetermine sample size (not applicable to data type). However, the sample size was sufficient to i) demonstrate that SVs captured expected population genetic structure observed in previous studies, ii) quantify statistically significant differences in SV allele frequency across populations, and iii) associate Atlantic salmon SVs with functional features in the genome. Therefore, the sample size was sufficient to provide reliable conclusions and novel biological insights on SVs, aligned to the original study aims. While the final number of samples sequenced was constrained by the available budget, this is by far the largest whole genome re-sequencing effort to date in Atlantic salmon, and as such it at least meets the criteria of what comprises what is a 'sufficient sample size' with respect to previously published studies.

Data collection

Wild Atlantic salmon were sampled during organized fishing expeditions or by anglers during the sport fishing season with scale samples taken for analysis. It is not possible to name every individual present during sampling due to the extensive scale of the effort, but sample collection was coordinated by expert co-authors based at the Norwegian Institute for Nature Research, Norway (Kjetil Hindar); Radgivende Biologer, Norway (Harald Sægrov); Norwegian Veterinary Institute, Norway (Bjørn Florø-Larsen); Natural Resources Institute, Finland (Jaakko Erkinaro) and the Université Laval (Louis Bernatchez); note that per sample information on coordinating institutes is provided in Supplementary Table 1. Information on sampling site and coordinates was collated in an Excel sheet, with the information provided in Supplementary Table 1. Farmed Atlantic salmon samples from Mowi were gifted as paired end sequencing data by Dr Serap Gonen, collected as part of the management of a commercial breeding programme. Farmed

	Atlantic salmon from Xelect represented muscle samples, gifted by co-author Professor Ian Johnston. The DNA sequencing library preparation and sequencing was done by commercial sequencing centres (not possible to list persons involved) using sequencing instruments provided on a per sample basis in Supplementary Table 1.
Timing and spatial scale	The rationale for the spatial scale of sampling and for sampling locations is described above in 'Research Sample'. Moreover, specific latitude-longitude coordinates of samples are given in Supplementary Table 1. The sequenced samples were collected between 2008 and 2017. The rationale for the timing of sample sequencing was based on when funding was available through grants listed in the paper. We stopped sequencing samples when the aim was achieved of having high quality sequencing data for multiple individuals per each of the major Atlantic salmon phylogeographic groups, including both wild and farmed populations.
Data exclusions	No data were excluded.
Reproducibility	This is not relevant to the main data type in the study used for SV detection, because genome re-sequencing data is not an experimental variable, rather a fixed observation per animal, with reproducibility achieved through sufficient sequencing coverage. Therefore, it is not usual to repeat whole genome sequencing of the same individuals more than once as the result is expected to be identical. The only relevant data type produced in the study is ATAC-Seq data, which was generated with four biological replicates, and only peaks detected in all four individuals were used in downstream analyses, ensuring these analyses are based on solely reproducible data. All analyses can be reproduced using the raw sequencing data provided in the study (see 'Data').
Randomization	Sampling was random, both for wild and farmed fish, within the populations targeted either in rivers for the given study sites, or within the populations of fish sampled from aquaculture stocks.
Blinding	No data was generated where blinding would affect the outcomes. There are no prior beliefs or expectations that could affect the outcome of the results reported.

Did the study involve field work? [x] Yes [ ] No

Field work, collection and transport

Field conditions	Not possible to provide - large sample numbers. Key information provided in Supplementary Table 1.
Location	Latitude and longitude for all wild fish sampled is provided in Supplementary Table 1.
Access & import/export	All field-collected samples used in sequencing were obtained by authorized organizations listed as co-authors on the manuscript, in full compliance with local regulations. Samples collected by Rådgivende Biologer (Norway) (Supplementary Table 1) were obtained as part of an annual scale sampling program in West Norwegian rivers that has been ongoing since 1999. This program is supported by the Norwegian Environment Agency, whom request that fishermen take scales from any Atlantic salmon they catch; no additional permissions were required. Samples obtained by the Natural Resources Institute (Finland) (Supplementary Table 1) were largely collected as part of yearly monitoring of the salmon populations, from legal catches made by anglers during the fishing season; no additional permissions were needed. The one exception is the Utsjoki (a tributary of the Tana river), where samples came from a research project led by co-author Craig Primmer, with permissions obtained from local and regional fisheries authorities (Utsjoki Fisheries Co-operative and ELY Centre for Lapland). Samples coordinated by Université Laval (Supplementary Table 1) were collected directly by (and under the authorization) of the local ministry of fisheries and wildlife and Ministère des Fôrets de la Faune et des Parcs du Québec; this did not require any specific permits. Samples obtained by the Norwegian Institute for Nature Research, Norway (Supplementary Table 1) were collected during a routine sampling program, with scales collected by anglers, forming part of routine population monitoring of wild Atlantic salmon in Norway, initiated by the Environment Agency of Norway and financed by the Environment Agency of Norway and the Research Council of Norway; no further permissions were required. Samples obtained from the Norwegian Veterinary Institute, Norway (Supplementary Table 1) were collected either from anglers during the summer sport fishing season supported by local fishing-rights owners, or from wild broodstock collected in the autumn, supported by the Norwegian Environment Agency, which requires sampling of all fish intended for use as broodstock; no further permissions were required.
Disturbance	Disturbance to wild fish was minimized where possible by sampling catches from fisherman that had caught the animals already or as part of routine monitoring of wild stocks by authorized organizations.

Reporting for specific materials, systems and methods

We require information from authors about some types of materials, experimental systems and methods used in many studies. Here, indicate whether each material, system or method listed is relevant to your study. If you are not sure if a list item applies to your research, read the appropriate section before selecting a response.

Materials & experimental systems

n/a	Involved in the study
x	Antibodies
x	Eukaryotic cell lines
x	Palaeontology and archaeology
	x Animals and other organisms
x	Human research participants
x	Clinical data
x	Dual use research of concern

Methods

n/a	Involved in the study
x	ChIP-seq
x	Flow cytometry
x	MRI-based neuroimaging

Animals and other organisms

Policy information about studies involving animals; ARRIVE guidelines recommended for reporting animal research

Laboratory animals	For ATAC-Seq study, n=4 farmed Atlantic salmon were used; (no strain information available), unsexed, age unknown, weight 26-28g
Wild animals	Mixed age and sex Atlantic salmon were caught and sampled in the field. All sampling performed was non-lethal, with no animals captured, transported or killed for purposes of the research.
Field-collected samples	The study did not involve laboratory work with field-collected samples.
Ethics oversight	Not applicable for genome sequencing samples, as no lab was work performed. For the ATAC-Seq study, the welfare and use of animals was done in strict accordance with the Norwegian Animal Welfare Act 2010 and animals were killed using a Schedule 1 method following the Animals (Scientific Procedures) Act 1986. No specific ethical approval was required due to the lack of experimental manipulation.

Note that full information on the approval of the study protocol must also be provided in the manuscript.
