# Supplementary material for: The structural variation landscape in 492 Atlantic salmon genomes
Source: Nat Commun. 2020 Oct 14;11:5176. doi: 10.1038/s41467-020-18972-x (PMC7560756; doi:10.1038/s41467-020-18972-x)
Supplement: Supplementary file 3 — Description of Additional Supplementary Files [file 41467_2020_18972_MOESM3_ESM.pdf]

Description of Additional Supplementary Files

Supplementary Data 1. Details of samples used in study

Supplementary Data 2. SV call statistics per individual across 492 Atlantic salmon samples following different filtering steps

Supplementary Data 3. Full SV dataset and genotypes prior to SV-plaudit curation

Supplementary Data 4. Validation of SV calls and genotypes using MinION sequencing

Supplementary Data 5. High-confidence SVs retained after SV-plaudit curation, including individual genotypes and SnpEff annotation

Supplementary Data 6. GO Biological Process enrichment analysis for genes affected by high impact deletions, duplications and inversions

Supplementary Data 7. Genes contributing to significant GO terms for high impact SVs

Supplementary Data 8. Alignment of SV deletions representing pTSsa2 piggyBac-like DNA transposons (used to Generate Fig. 2)

Supplementary Data 9. High confidence annotation of Ss4R ohnolog and singletons in the Atlantic salmon genome

Supplementary Data 10. Fishers Exact test results contrasting the overlap between SVs with singleton genes vs. Ss4R ohnolog genes.

Supplementary Data 11. Manually filtered SV deletions that alter protein-coding exons

Supplementary Data 12. Significant SV outliers between wild and farmed salmon from Norway

Supplementary Data 13. GO enrichment analysis for genes linked to SV outliers between wild and farmed Atlantic salmon

Supplementary Data 14. Genes contributing to significant GO terms for genes linked to SV outliers.

Supplementary Data 15. Statistical tests of two expression characteristics (specificity and level) across a panel of tissues for 327 SV outlier linked genes contributing to significantly enriched GO biological processes in comparison to a transcriptome-wide set gene set.

Supplementary Data 16. Detailed annotation of prioritized SV outliers between farmed and wild Atlantic salmon linked to genes with synaptic functions.

Supplementary Data 17-19: Zip files for Gitlab repositories described in the Code Availability section
