# Supplementary figures and images for: The structural variation landscape in 492 Atlantic salmon genomes
Source: Nat Commun. 2020 Oct 14;11:5176. doi: 10.1038/s41467-020-18972-x (PMC7560756; doi:10.1038/s41467-020-18972-x)

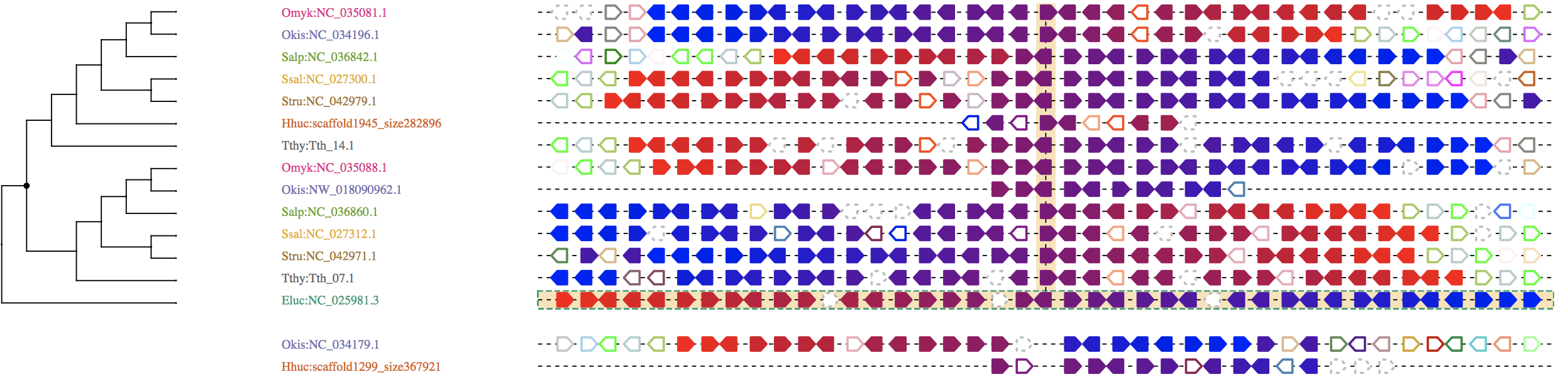

Supplement: Supplementary file 20 — Supplementary Data 17 [file 41467_2020_18972_MOESM20_ESM.zip › salmonid_synteny-master/synteny.png]

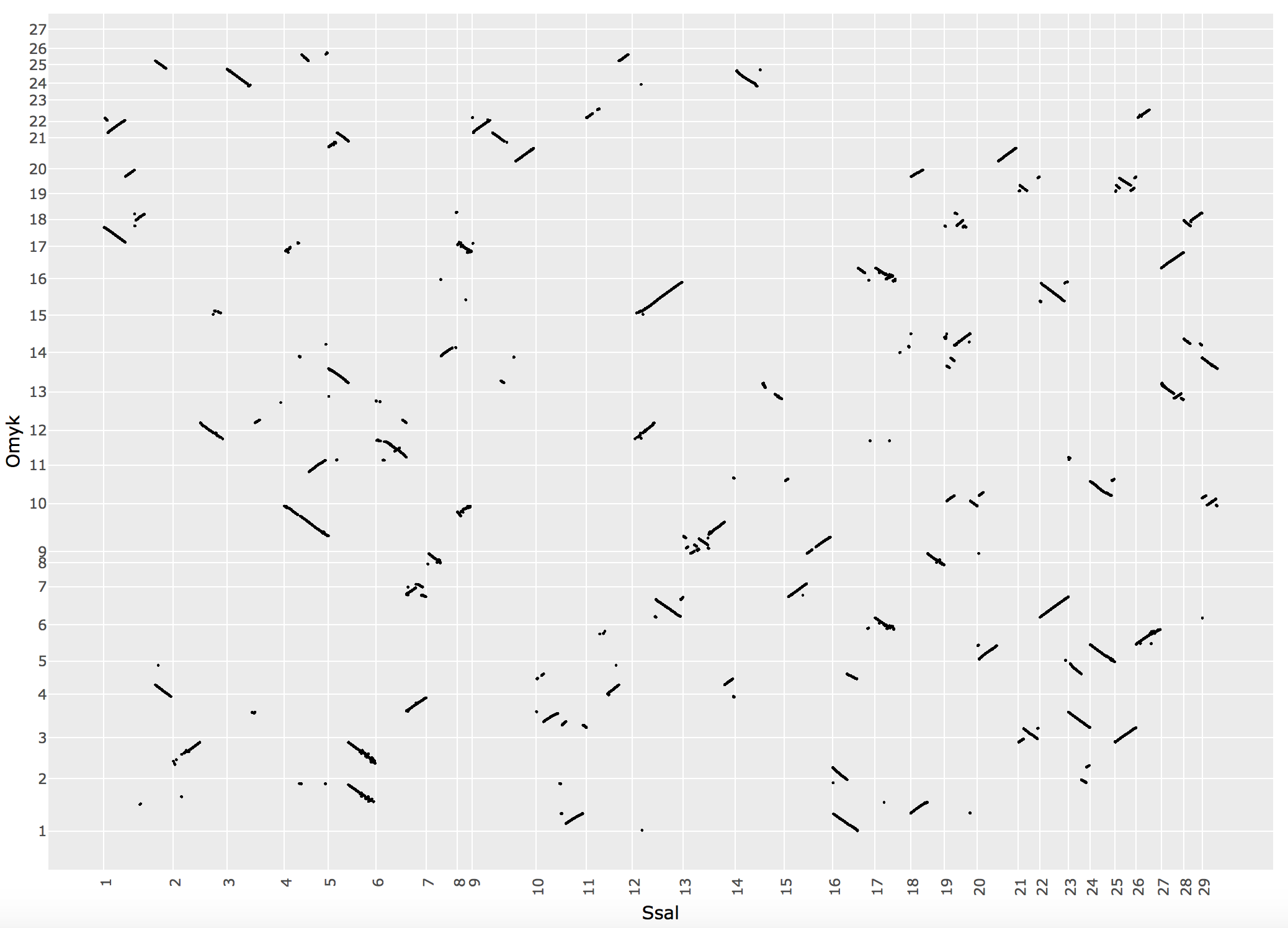

Supplement: Supplementary file 20 — Supplementary Data 17 [file 41467_2020_18972_MOESM20_ESM.zip › salmonid_synteny-master/dotplot2.png]

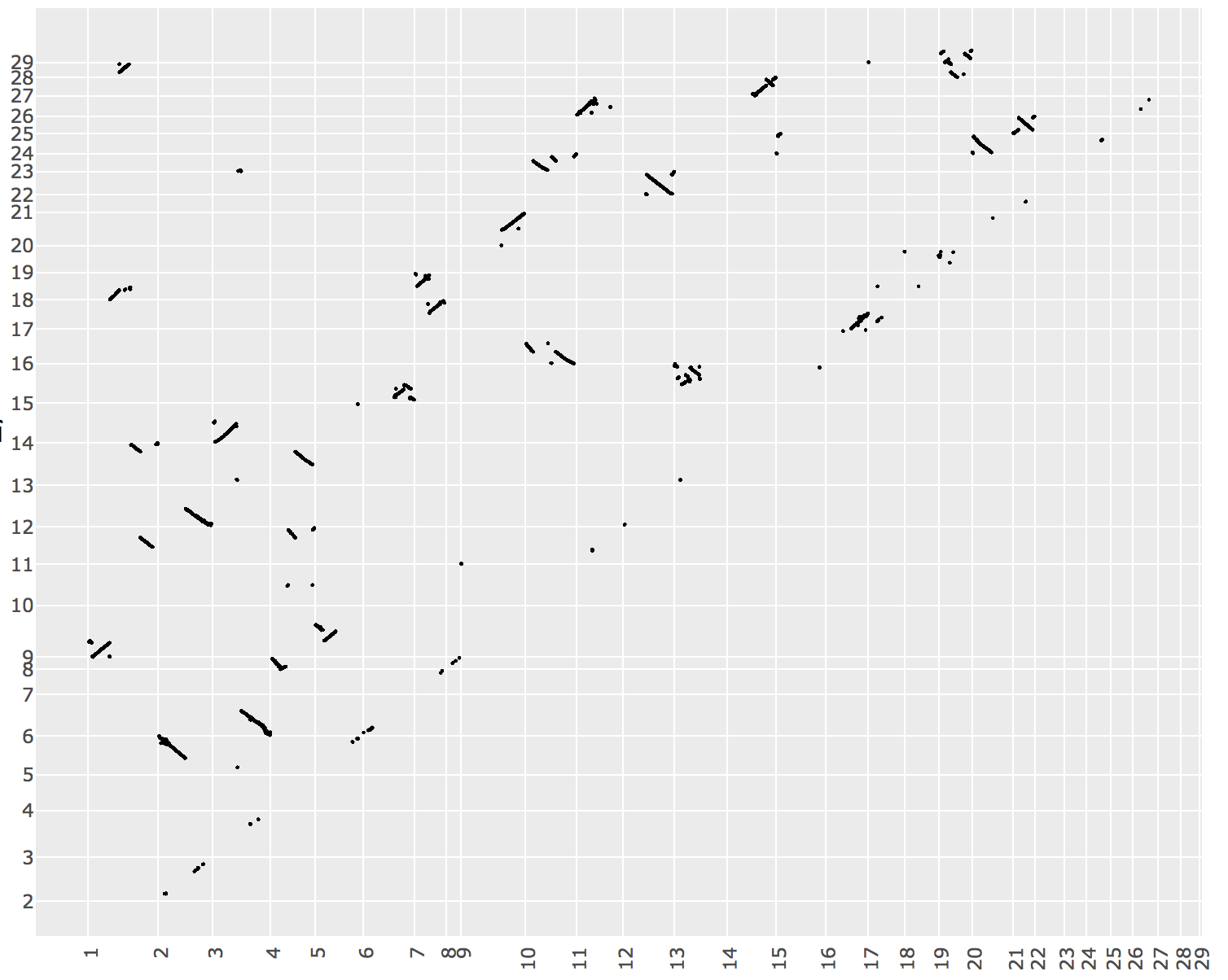

Supplement: Supplementary file 20 — Supplementary Data 17 [file 41467_2020_18972_MOESM20_ESM.zip › salmonid_synteny-master/dotplot1.png]

**ALL SV types**  
affected pairs=1998

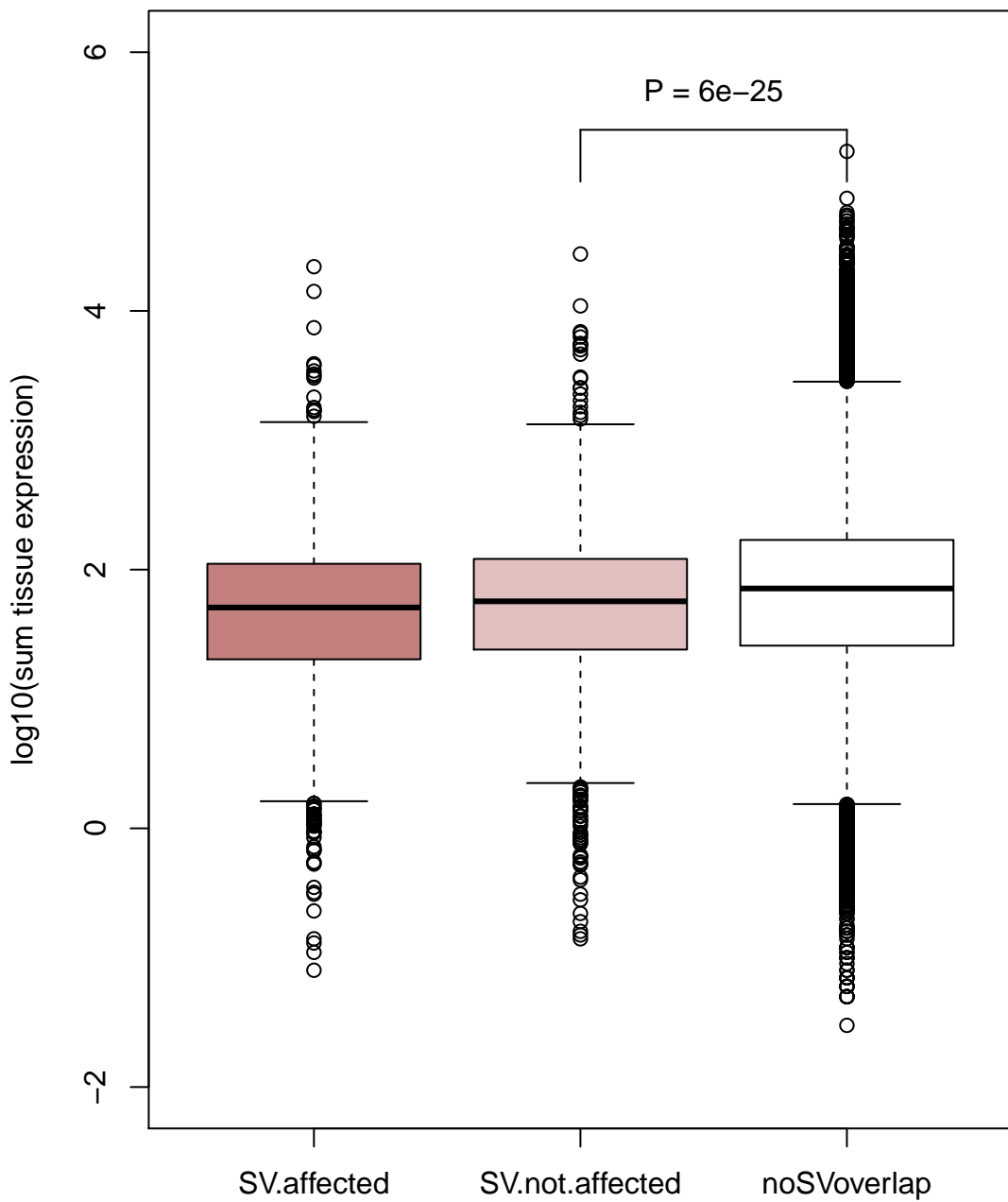

**DEL SV types**  
affected pairs =1852

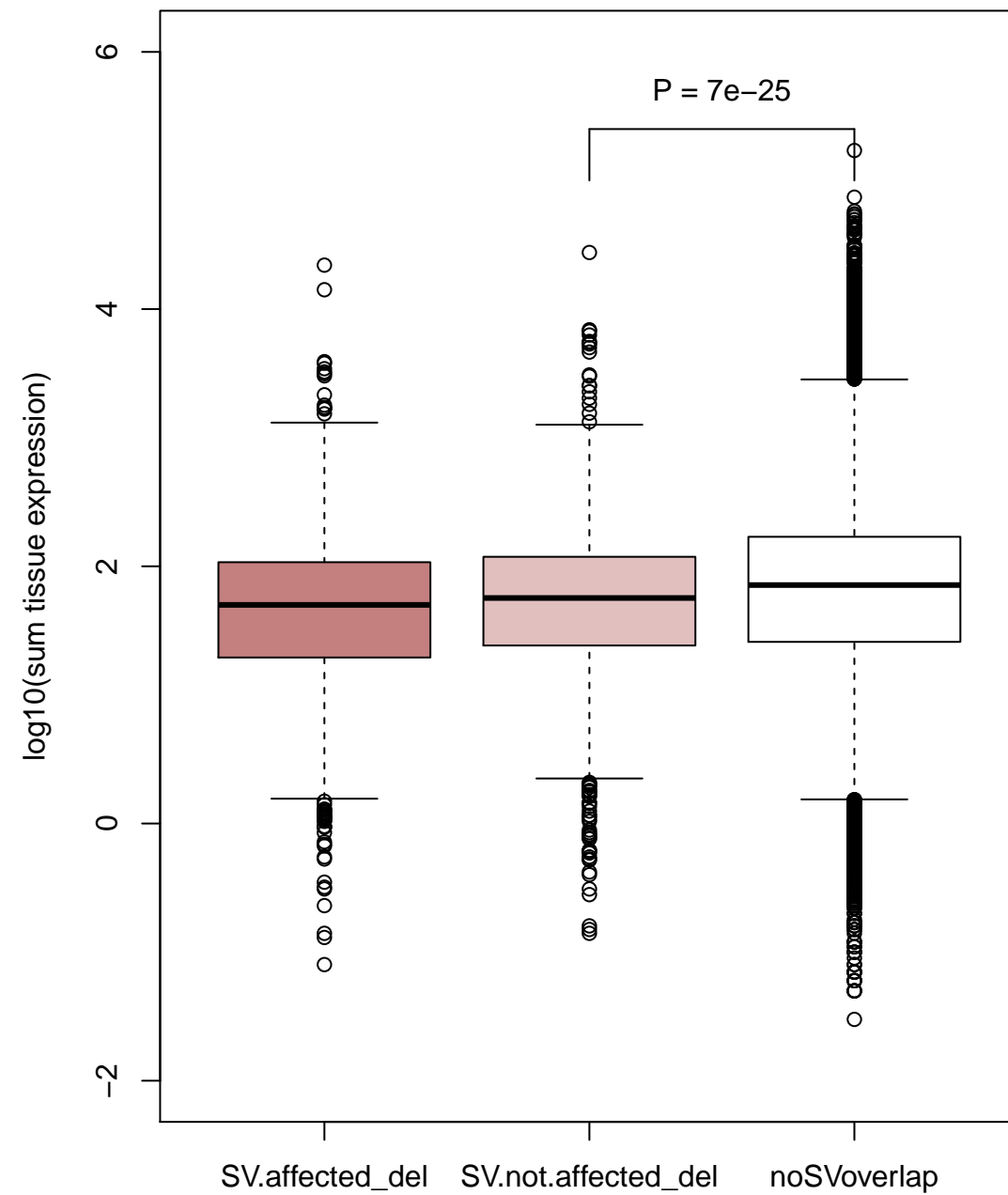

Supplement: Supplementary file 21 — Supplementary Data 18 [file 41467_2020_18972_MOESM21_ESM.zip › atlantic_salmon_sv_ohnolog_analyses-master/results/boxplot_expression_levels.pdf]

# Resampled spearman corrs.

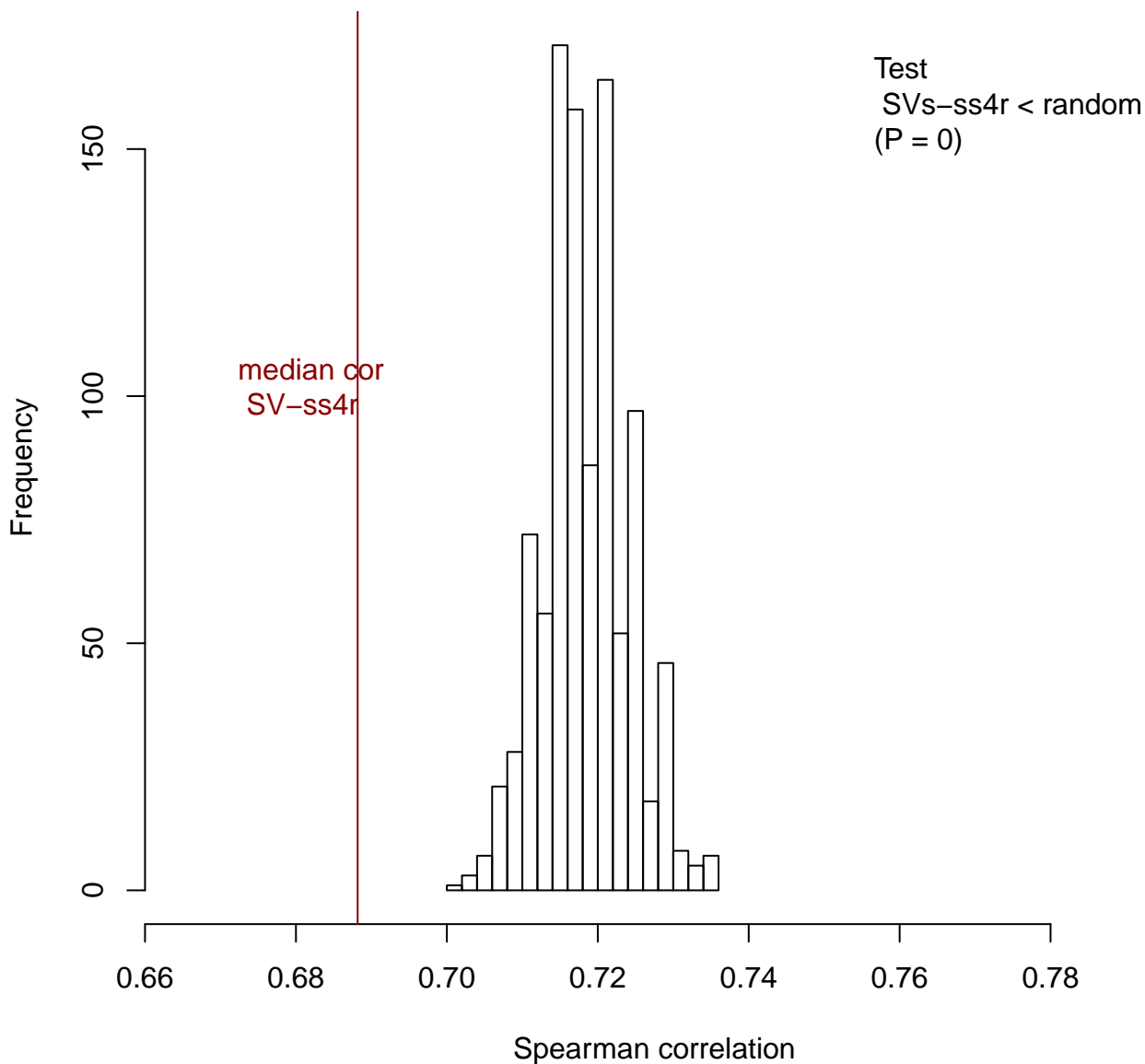

Supplement: Supplementary file 21 — Supplementary Data 18 [file 41467_2020_18972_MOESM21_ESM.zip › atlantic_salmon_sv_ohnolog_analyses-master/results/resample_expression_correlation.pdf]
