# Supplementary material for: The structural variation landscape in 492 Atlantic salmon genomes
Source: Nat Commun. 2020 Oct 14;11:5176. doi: 10.1038/s41467-020-18972-x (PMC7560756; doi:10.1038/s41467-020-18972-x)
Supplement: Supplementary file 21 — Supplementary Data 18 [file 41467_2020_18972_MOESM21_ESM.zip › atlantic_salmon_sv_ohnolog_analyses-master/SVs_DUPs_and_ATAC.html]

SV analyses - DUPs and ATAC


Code 

- Show All Code
- Hide All Code

# SV analyses - DUPs and ATAC

#### Simen & Teshome

#### spring 2020

- The data:
- Removing redundancy in the SV features
- Make a list of SVs that overlap genes
- Overlap between ss4R dups and SVs
  - Only SVs overlapping sinlge genes
  - SVs overlapping any number of genes
- Does domestication act on Ss4Rs ?
  - Association between SV-ssr4 overlap and divergence in expression levels
- SV overlap with ATAC

**The document contains the following analyses:**

- Analyses of Ss4R duplicates and their propensity to be tagged by an SV

## The data:

In the following analyses we use the `Supplementary_data_2` data.

```
SuppData2 <- read_tsv("data/Supplementary_data_2.txt")
```

```
## Parsed with column specification:
## cols(
##   .default = col_double(),
##   Chromosome = col_character(),
##   SV_class = col_character(),
##   SnpEff_annotation = col_character(),
##   SnpEff_impact = col_character(),
##   `Associated_gene/s` = col_character()
## )
```

```
## See spec(...) for full column specifications.
```

```
SuppData6 <- read_tsv("data/Supplementary_data_6.txt")
```

```
## Parsed with column specification:
## cols(
##   .default = col_double(),
##   UniqueID = col_character(),
##   Chromosome = col_character(),
##   Type = col_character(),
##   SnpEffeffect = col_character(),
##   SnpEffImpact = col_character(),
##   AffectedGene = col_character(),
##   `FstFvW(Canada)` = col_character()
## )
## See spec(...) for full column specifications.
```

```
unique.sup6 <- paste(SuppData6$Chromosome, SuppData6$Start, SuppData6$End, sep = '_')
unique.sup2 <- paste(SuppData2$Chromosome, SuppData2$Start, SuppData2$End, sep='_')
SuppData2$FST <- SuppData6$UniqueID[match(unique.sup2, unique.sup6)]

SVs <- data.frame(unique.sup2, select(SuppData2, c(1:8, 502)))
SVs <- as_tibble(SVs)
colnames(SVs) <- c('unique.ID', 'chrom', 'start', 'end', 'size.bp', 'type', 'snpeff.annot', 'snpeff.impact', 'closest.gene', 'FST')
SVs$start <- as.integer(SVs$start)
SVs$end <- as.integer(SVs$end)
```

This file contains the full list of validated SVs (15616). There are 3 diffreent types of SVs definded:

```
table(SVs$type)
```

```
## 
## <DEL> <DUP> <INV> 
## 14056  1251   309
```

## Removing redundancy in the SV features

By inspecting the data I found that different SV-features overlapped eachoter. This likely represent redundancy in the SV calling and should possibly be removed from the data. See example below..where three SV features overlap extensively and is likely the same SV.

Therefore to make a non-redundant set of SVs we used `bedtools intersect` to identify SVs that overlapped other SVs.

```
# using bedttol to intersect features...

SVs <- SVs %>% arrange(chrom, start)
write_tsv(SVs[,c(2:4,1)], path = "data/SVs_bedformat.txt", col_names = F)


system('bedtools intersect -wa -wb -a /Users/srsand/Dropbox/Work/Projects/SVs_Dan_Teshome/atlantic_salmon_sv_ohnolog_analyses/data/SVs_bedformat.txt -b /Users/srsand/Dropbox/Work/Projects/SVs_Dan_Teshome/atlantic_salmon_sv_ohnolog_analyses/data/SVs_bedformat.txt -sorted > /Users/srsand/Dropbox/Work/Projects/SVs_Dan_Teshome/atlantic_salmon_sv_ohnolog_analyses/results/SVs_overlap.txt')

system('bedtools intersect -a /Users/srsand/Dropbox/Work/Projects/SVs_Dan_Teshome/atlantic_salmon_sv_ohnolog_analyses/data/SVs_bedformat.txt -b /Users/srsand/Dropbox/Work/Projects/SVs_Dan_Teshome/atlantic_salmon_sv_ohnolog_analyses/data/SVs_bedformat.txt -sorted -c > /Users/srsand/Dropbox/Work/Projects/SVs_Dan_Teshome/atlantic_salmon_sv_ohnolog_analyses/results/number_SVs_overlap.txt')

SV_intersectresults <- read_tsv("results/SVs_overlap.txt", col_names = F)
```

```
## Parsed with column specification:
## cols(
##   X1 = col_character(),
##   X2 = col_double(),
##   X3 = col_double(),
##   X4 = col_character(),
##   X5 = col_character(),
##   X6 = col_double(),
##   X7 = col_double(),
##   X8 = col_character()
## )
```

```
SV_overlapnumber <- read_tsv("results/number_SVs_overlap.txt", col_names = F)
```

```
## Parsed with column specification:
## cols(
##   X1 = col_character(),
##   X2 = col_double(),
##   X3 = col_double(),
##   X4 = col_character(),
##   X5 = col_double()
## )
```

```
# make some plots
par(mfrow=c(1,3))
barplot(table((SV_overlapnumber$X5-1))[-1], main="Overlapping SV features to other SVs", xlab="Number of overlaps with non-self (e.g. other) SV features")

SV_intersectresults$length_a <- SV_intersectresults$X3-SV_intersectresults$X2
SV_intersectresults <- SV_intersectresults %>% arrange(desc(length_a)) 

redundant_length <- filter(SVs, SVs$unique.ID %in% filter(SV_overlapnumber, X5>1)$X4) %>% mutate(SV.length=end-start) %>% select(SV.length)

NONredundant_length <- filter(SVs, SVs$unique.ID %in% filter(SV_overlapnumber, X5==1)$X4) %>% mutate(SV.length=end-start) %>% select(SV.length)

boxplot(list(redundant=redundant_length$SV.length, NONredundant=NONredundant_length$SV.length), outline=F, ylab='length SV bp')

barplot(table(filter(SVs, SVs$unique.ID %in% filter(SV_overlapnumber, X5>1)$X4)$type), ylab="Number of SVs", xlab="class of SV")
```

```
SVs_nooverlap <- filter(SVs, !unique.ID %in% SV_intersectresults$X8[which(duplicated(SV_intersectresults$X4))])
SVs_removed_overlapping <- SVs$unique.ID[! SVs$unique.ID %in% SVs_nooverlap$unique.ID]

SVs <- SVs_nooverlap


# write results to bed
writeLines(as.character(SVs_removed_overlapping), con = 'results/SVs_removed_redundant.txt')
SVs_nooverlap_bed <- SVs_nooverlap %>% select(chrom, start, end, unique.ID)
write_tsv(SVs_nooverlap_bed, path = 'results/SVs_redundancyfiltered.bed', col_names = F)
```

Finally we made a non-redundant set of SV calls. This data contains 14938 SVs. For each SV that overlapped one or more other SV features, we retained the longest SV.

## Make a list of SVs that overlap genes

For each SV-feature the FST between wild and aquaculture salmon has been calculated.

By intersecting the 14938 SV features with genes from the NCBI RefSeq annotation we identified 7365 SVs overlpapping one or more genes. In total, 8349 genes were overlapped by an SV. When an SV overlapped a gene, the most common case was overlap of a single gene (7043 SVs), however the most extreme overlapped 51 genes.

```
par(mfrow=c(1,1))
nb.overlapgenes <- SVs$nb.overlapgenes
nb.overlapgenes[nb.overlapgenes>10]<-10
barplot(table(nb.overlapgenes)[-1], names.arg = c(1:9, '>10'), xlab = 'No. genes overlapped (including UTRs, CDS, introns)', ylab = 'Number of Svs')
```

## Overlap between ss4R dups and SVs

To test the idea that many ss4r dups evolve under neutral evolution and can thus tolerate more mutations, including being incvolved in more SVs, we counted how many SVs (and what type) overlapped ss4r genes. We started with SVs overlapping one gene only.

```
# all dups and singletons in one table
dups_gather <- data.frame(type=DUPSINtbl$type, gene_id = DUPSINtbl$gene1)
gene2 <- na.omit(DUPSINtbl$gene2)
dups_gather <- rbind(dups_gather, data.frame(type='ss4r', gene_id=gene2))
dups_gather <- dups_gather[!duplicated(dups_gather$gene_id),]
```

### Only SVs overlapping sinlge genes

```
# overlap test

## check this...

test_overlaps = function(overlaps.testset=overlapgenes_all$overlapgenes, universe=dups_gather){
    SV_overlap <- gsub('.*\\:|\")', '', unlist(strsplit(as.character(overlaps.testset), split = ';')))
    tab_SV <- table(universe$type[universe$gene_id %in% SV_overlap])
    tab_rest <- universe[! universe$gene_id %in% overlaps.testset,]
    tab_rest <- table(tab_rest$type)  
    test.tab = rbind(tab_rest, tab_SV)
    fish.test <- fisher.test(test.tab)    
    l = list(test.tab, fish.test)
    names(l) <- c('tab', 'res')
    l
}

# making results table
make_table = function(tab = test.tab.SVoverlap_any, fish.res = fish.test_any, rowname=''){
        df = data.frame(single.SV=tab[2,1], dup.SV =tab[2,2], single.No.SV=tab[1,1], dup.No.SV = tab[1,2], p.value=fish.res$p.value, oddsratio = round(fish.res$estimate, 2), CI = paste(round(fish.res$conf.int,2), collapse = '-'))
        rownames(df) <- rowname
      df        
}


SV_geneoverlap <- SVs %>% filter(nb.overlapgenes == 1) %>% select(overlapgenes)
SV_geneoverlap_DEL <- SVs %>% filter(type=='<DEL>', nb.overlapgenes == 1) %>% select(overlapgenes)
SV_geneoverlap_DUP <- SVs %>% filter(type=='<DUP>', nb.overlapgenes == 1) %>% select(overlapgenes)
SV_geneoverlap_HIGH <- SVs %>% filter(snpeff.impact == 'HIGH' , nb.overlapgenes == 1) %>% select(overlapgenes)
SV_geneoverlap_HIGH_IMPACT <- SVs %>% filter(high_impact==T, nb.overlapgenes == 1) %>% select(overlapgenes)


SV =  test_overlaps(SV_geneoverlap$overlapgenes, dups_gather)
SV_dup = test_overlaps(SV_geneoverlap_DUP$overlapgenes, dups_gather)
SV_del = test_overlaps(SV_geneoverlap_DEL$overlapgenes, dups_gather)
SV_high = test_overlaps(SV_geneoverlap_HIGH$overlapgenes, dups_gather)
SV_high_impacts = test_overlaps(SV_geneoverlap_HIGH_IMPACT$overlapgenes, dups_gather)

tab_single <- rbind(make_table(SV$tab, SV$res , rowname = 'All_SVs'),
      make_table(SV_del$tab, SV_del$res, rowname = 'DEL_SVs'),
      make_table(SV_dup$tab, SV_dup$res , rowname = 'DUP_SVs'),
      make_table(SV_high$tab, SV_high$res, rowname = 'HIGH_impact_snpeff_SVs'),
      make_table(SV_high_impacts$tab, SV_high_impacts$res, rowname = 'HIGH_impact_DAN_curation_SVs'))

percent_dups_overlapped_single <- round(tab_single$dup.SV/(tab_single$single.SV+tab_single$dup.SV), 3)*100    
write_tsv(tab_single, 'results/Results_SV_overlap_singlegenes_fisher.txt', col_names = T)
tab_single
```

```
##                              single.SV dup.SV single.No.SV dup.No.SV
## All_SVs                            855   3074         7427     16972
## DEL_SVs                            781   2884         7501     17162
## DUP_SVs                            101    281         8181     19765
## HIGH_impact_snpeff_SVs              97    245         8185     19801
## HIGH_impact_DAN_curation_SVs        42    115         8240     19931
##                                   p.value oddsratio        CI
## All_SVs                      6.191447e-30      1.57 1.45-1.71
## DEL_SVs                      4.465593e-31      1.61 1.48-1.76
## DUP_SVs                      2.347773e-01      1.15 0.91-1.46
## HIGH_impact_snpeff_SVs       7.650019e-01      1.04 0.82-1.34
## HIGH_impact_DAN_curation_SVs 5.384462e-01      1.13 0.79-1.65
```

The results coearly shows that all-SVs and DEL-SVs overlap more (78.2 and 78.7), compared to the other SV-categories (71.6 - 73.6).

### SVs overlapping any number of genes

```
overlapgenes_all <- SVs %>% filter(nb.overlapgenes >0) %>% select(overlapgenes)
overlapgenes_DUP_all <- SVs %>% filter(type == '<DUP>', nb.overlapgenes > 0) %>% select(overlapgenes)
overlapgenes_DEL_all <- SVs %>% filter(type%in%'<DEL>', nb.overlapgenes > 0) %>% select(overlapgenes)
overlapgenes_INV_all <- SVs %>% filter(type == '<INV>', nb.overlapgenes > 0) %>% select(overlapgenes)
overlapgenes_HIGH_all  <- SVs %>% filter(snpeff.impact == 'HIGH' , nb.overlapgenes >0) %>% select(overlapgenes)
overlapgenes_HIGH_IMPACT_all <- SVs %>% filter(high_impact==T, nb.overlapgenes > 0) %>% select(overlapgenes)

SV_all =  test_overlaps(overlapgenes_all$overlapgenes, dups_gather)
SV_all_dup = test_overlaps(overlapgenes_DUP_all$overlapgenes, dups_gather)
SV_all_del = test_overlaps(overlapgenes_DEL_all$overlapgenes, dups_gather)
SV_all_inv = test_overlaps(overlapgenes_INV_all$overlapgenes, dups_gather)
SV_all_high = test_overlaps(overlapgenes_HIGH_all$overlapgenes, dups_gather)
SV_all_high_impact = test_overlaps(overlapgenes_HIGH_IMPACT_all$overlapgenes, dups_gather)

## making results table:
tab_all <- rbind(make_table(SV_all$tab , SV_all$res , rowname = 'All_SVs'),
      make_table(SV_all_del$tab , SV_all_del$res , rowname = 'DEL_SVs'),
      make_table(SV_all_dup$tab , SV_all_dup$res, rowname = 'DUP_SVs'),
      make_table(SV_all_inv$tab , SV_all_inv$res, rowname = 'INV_SVs'),
      make_table(SV_all_high$tab , SV_all_high$res , rowname = 'HIGH_impact_snpeff_SVs'),
      make_table(SV_all_high_impact$tab , SV_all_high_impact$res , rowname = 'HIGH_impact_DAN_curation_SVs'))

write_tsv(tab_all, 'results/Results_SV_overlap_allgenes_fisher.txt', col_names = T)

percent_dups_overlapped <- round(tab_all$dup.SV/(tab_all$single.SV+tab_all$dup.SV), 3)*100
tab_all
```

```
##                              single.SV dup.SV single.No.SV dup.No.SV
## All_SVs                           1060   3557         7427     16972
## DEL_SVs                            809   2994         7501     17162
## DUP_SVs                            146    371         8181     19765
## INV_SVs                            152    347         8272     19999
## HIGH_impact_snpeff_SVs             232    558         8185     19801
## HIGH_impact_DAN_curation_SVs        46    125         8240     19931
##                                   p.value oddsratio        CI
## All_SVs                      1.877537e-25      1.47 1.36-1.58
## DEL_SVs                      2.545504e-32      1.62 1.49-1.76
## DUP_SVs                      6.259854e-01      1.05 0.86-1.28
## INV_SVs                      5.519003e-01      0.94 0.78-1.15
## HIGH_impact_snpeff_SVs       9.368193e-01      0.99 0.85-1.17
## HIGH_impact_DAN_curation_SVs 5.551981e-01      1.12 0.79-1.61
```

The results coearly shows that all-SVs and DEL-SVs overlap more (77 and 78.7), compared to the other SV-categories (69.5 - 71.8).

## Does domestication act on Ss4Rs ?

```
SVs_fst <- SVs[!is.na(SVs$FST),] # removing SVs not included in FST test
SVs_fst$FST <- gsub('lier-.*', '', SVs_fst$FST)

SVs_fst_low  <- SVs_fst %>% filter(FST == 'Non-out') %>% select(overlapgenes)
SVs_fst_high <- SVs_fst %>% filter(FST == 'Out') %>% select(overlapgenes)

dups_gather_lowfst <- dups_gather %>% filter(gene_id %in% gsub('.*\\:', '', unlist(strsplit(na.omit(SVs_fst_low$overlapgenes), ';'))))

dups_gather_highfst <- dups_gather %>% filter(gene_id %in% gsub('.*\\:', '', unlist(strsplit(na.omit(SVs_fst_high$overlapgenes), ';'))))

test.table.ss4r_fstgroups <- rbind(table(dups_gather_lowfst$type), table(dups_gather_highfst$type))
rownames(test.table.ss4r_fstgroups) <- c('Non-outliers', 'Outliers')
test.ss4r_fstgroups = fisher.test(test.table.ss4r_fstgroups)


percent_dupoverlap_fst <- test.table.ss4r_fstgroups[,2]/(test.table.ss4r_fstgroups[,2]+test.table.ss4r_fstgroups[,1])
percent_dupoverlap_fst <- round(percent_dupoverlap_fst, 3)*100
test.table.ss4r_fstgroups
```

```
##              singleton ss4r
## Non-outliers       882 2886
## Outliers            45  211
```

To check if the SVs with different allele frequency in domestic and wild were enriched in Ss4R (hypothesis: dups are targets for domestication bottlenecks/selection) we used a fisher test to compare distributions of singletons/dups in SVs with high (outliers) vs low FST values (non-outliers). These distributinos were not identical (p-value = 0.03, proportion outliers overlapping dups = 82.4%, proportion non-outliers overlapping dups = 76.6%, suggesting a slight bias in allele frequency shift for SVs that overlap Ss4R duplicates.

### Association between SV-ssr4 overlap and divergence in expression levels

#### Dup-pairs where one copy is overlapped by an SV has *decreased correlation*

```
# Subset the ss4r table to ohnologs where one has a deletion and the other has 'nothing'
ss4r <- filter(DUPSINtbl, type=='ss4r')

ss4r$tagged_by_SV_1 <- ss4r$gene1 %in% SVs$overlapgenes
ss4r$tagged_by_SV_2 <- ss4r$gene2 %in% SVs$overlapgenes

table_dupoverlap <- table(paste(ss4r$tagged_by_SV_1, ss4r$tagged_by_SV_2))
table_dupoverlap[2] <- table_dupoverlap[2] + table_dupoverlap[3]
table_dupoverlap <- table_dupoverlap[-3]
names(table_dupoverlap) <- c('none overlap', 'one overlap', 'both overlapped')
ss4r$idx.oneSV <- paste(ss4r$tagged_by_SV_1, ss4r$tagged_by_SV_2) %in% c('FALSE TRUE', 'TRUE FALSE')


# for each pair of DUPS, calculate spearman correlation distance for tissue expression
tissuepanel_raw <- salmonfisher::fish_expression()
tissuepanel_raw <- tissuepanel_raw[!is.na(rowSums(tissuepanel_raw[,-1])),] # remove genes with NA observations in gene expressino table & sd == 0
tissuepanel_raw <- tissuepanel_raw[which(rowSums(tissuepanel_raw[,-1]) > 0), ] # remove genes with zero expression


## duplicate divergence : correlation
expr.sums <- data.frame(gene_id = tissuepanel_raw$gene_id, sum=rowSums(tissuepanel_raw[,-1]))
ss4r$sum_dup1 <- expr.sums$sum[match(ss4r$gene1, expr.sums$gene_id)]
ss4r$sum_dup2 <- expr.sums$sum[match(ss4r$gene2, expr.sums$gene_id)]

dup.dist = c()

for(i in 1:nrow(ss4r)){
  #print(i)
  
  # cor dist
  idx1 <- which(tissuepanel_raw$gene_id %in% ss4r$gene1[i])
  idx2 <- which(tissuepanel_raw$gene_id %in% ss4r$gene2[i])
  
  if(length(c(idx1, idx2))!=2) {dup.dist[i] <- NA}
  if(length(c(idx1, idx2))==2){
  dup.dist[i] <- cor(as.numeric(tissuepanel_raw[idx1,-1]), as.numeric(tissuepanel_raw[idx2,-1]), method = 'spearman', use = "pairwise.complete.obs")
  }
  
}

ss4r$dup.dist <- dup.dist

# make plot of expression level difference of ohnologs affected with SV minus the other one...

# produce Plot for correlatoin resampling 
resamp_dist <- unlist(lapply(1:1000, function(i) median(sample(ss4r$dup.dist, sum(ss4r$idx.oneSV), replace = F), na.rm=T)))
# max(unlist(resamp_dist))
# min(unlist(resamp_dist))
resamp.p <- sum(unlist(resamp_dist) <=  median(ss4r$dup.dist[ss4r$idx.oneSV],na.rm = T))/1000
observed.corr.median <- median(ss4r$dup.dist[ss4r$idx.oneSV], na.rm = T)


par(mfrow=c(1,1))
hist(resamp_dist, breaks = 20, main="Resampled spearman corrs.", xlab = 'Spearman correlation', xlim = c(0.665, 0.79))
abline(v=observed.corr.median, col='darkred')
text(x=0.67, y=100, labels = 'median cor\n SV-ss4r ', col='darkred', pos = 4)
legend('topright', bty='n', legend = paste0("Test\n SVs-ss4r < random \n(P = ", resamp.p, ")"))
```

```
pdf('results/resample_expression_correlation.pdf')
par(mfrow=c(1,1))
hist(resamp_dist, breaks = 20, main="Resampled spearman corrs.", xlab = 'Spearman correlation', xlim = c(0.665, 0.79))
abline(v=observed.corr.median, col='darkred')
text(x=0.67, y=100, labels = 'median cor\n SV-ss4r ', col='darkred', pos = 4)
legend('topright', bty='n', legend = paste0("Test\n SVs-ss4r < random \n(P = ", resamp.p, ")"))
dev.off()
```

```
## quartz_off_screen 
##                 2
```

We tested if duplicate pairs (*one copy was overlapped by an SV and its ohnolog partner was not*) were particurarly divergent in tissue expression. We first calculated correlation (spearman) distances between all duplicate pairs across 15 tissues (RNA-seq) and performed a resampling test to test if dups affected by SVs also had diverged more in tissue expression regulation. Indeed, the median expression correlation between dups where ONE copy overlapped with an SV was significatly lower (corr = 0.6881765) compared to random pairs of ohnologs (resampling P-value = 0).

#### Expression levels are lower for ss4r genes associated with SVs

```
dup.sum.list = sapply(1:nrow(ss4r),function(x) NULL)
idx.overlapped = c()
for(i in 1:nrow(ss4r)){ # 
  #print(i)
  if(ss4r$idx.oneSV[i] %in% F) { dup.sum.list[[i]] <- NULL }
  if(ss4r$idx.oneSV[i] %in% T) { 
    idx.overlapped <- which(c(ss4r$tagged_by_SV_1[i], ss4r$tagged_by_SV_2[i]))
      if(idx.overlapped == 1) { dup.sum.list[[i]] <- c(ss4r$sum_dup2[i], ss4r$sum_dup1[i])}
      if(idx.overlapped == 2) { dup.sum.list[[i]] <- c(ss4r$sum_dup1[i], ss4r$sum_dup2[i]) }
  }
}

dup.sum.list_for_del <- dup.sum.list
dup.sum.list <- dup.sum.list %>% discard(is.null)

wilcox.diff = wilcox.test(x = sapply(dup.sum.list, '[[', 1), y = sapply(dup.sum.list, '[[', 2), paired = T, alternative = 'greater')
```

Expression levels are positively correlated with purifying selection pressure. We therefore tested if ohnologs overlapped by SVs have lower expression levels. We used a paired wilcoxon test to see if (within ohnolog oairs) SVs overlapped the copy with lowest expression. This was the case (P = 2.910^{-6}).

We then extracted the expression levels (sum across all tissues) and plotted this for ss4r pairs (red colour) for which one copy was overlapped by an SV, and compared it to ohnologs where none of the dups was tagged by an SV. We did this both for all types of SVs (first figure), and then only deletions.

```
##
log.ohnolog_SV_notaffected <- log10(sapply(dup.sum.list, '[[', 1))
log.ohnolog_SV_affected <- log10(sapply(dup.sum.list, '[[', 2))
ohnolog_noSV <- c(ss4r$sum_dup1[paste(ss4r$tagged_by_SV_1, ss4r$tagged_by_SV_2) %in% 'FALSE FALSE'],
  ss4r$sum_dup2[paste(ss4r$tagged_by_SV_1, ss4r$tagged_by_SV_2) %in% 'FALSE FALSE'])
log.ohnolog_noSV <- log10(ohnolog_noSV)

par(mfrow=c(1,2))
boxplot(list(SV.affected = log.ohnolog_SV_affected, 
             SV.not.affected = log.ohnolog_SV_notaffected,
             noSVoverlap = log.ohnolog_noSV), 
            col=c(alpha('darkred', 0.5), alpha('darkred', 0.25), 'white'), ylab='log10(sum tissue expression)', ylim=c(-2,6), main=paste0('ALL SV types\n', 'affected pairs=',length(log.ohnolog_SV_notaffected)))

wilcox.test.all <- wilcox.test(sapply(dup.sum.list, '[[', 2),
              ohnolog_noSV, 'less')

segments(x0=2, x1=3, y0=5.4)
segments(y0=5, y1=5.4, x0=2)
segments(y0=5, y1=5.4, x0=3)
text(x=2.5, y=5.7, labels = paste0('P = ', round(wilcox.test.all$p.value, 25)))


# identifying which pairs from dup_sum_list that represents deletions only...

del.overlapgenes <- SVs %>% filter(type == "<DEL>") %>% select(overlapgenes) 
gene1_deloverlap = ss4r$gene1 %in% del.overlapgenes$overlapgenes
gene2_deloverlap = ss4r$gene2 %in% del.overlapgenes$overlapgenes
idx.del.overlaps_oneSV <- which((gene1_deloverlap | gene2_deloverlap)  & ss4r$idx.oneSV) # 
dup.sum.list_for_del_filt <- dup.sum.list_for_del[idx.del.overlaps_oneSV]

log.ohnolog_SV_notaffected_del <- log10(sapply(dup.sum.list_for_del_filt, '[[', 1))
log.ohnolog_SV_affected_del <- log10(sapply(dup.sum.list_for_del_filt, '[[', 2))
ohnolog_noSV <- c(ss4r$sum_dup1[paste(ss4r$tagged_by_SV_1, ss4r$tagged_by_SV_2) %in% 'FALSE FALSE'],
                  ss4r$sum_dup2[paste(ss4r$tagged_by_SV_1, ss4r$tagged_by_SV_2) %in% 'FALSE FALSE'])
log.ohnolog_noSV <- log10(ohnolog_noSV)

boxplot(list(SV.affected_del = log.ohnolog_SV_affected_del, 
             SV.not.affected_del = log.ohnolog_SV_notaffected_del,
             noSVoverlap = log.ohnolog_noSV), 
            col=c(alpha('darkred', 0.5), alpha('darkred', 0.25), 'white'), ylab='log10(sum tissue expression)', ylim=c(-2, 6), main=paste0('DEL SV types\n', 'affected pairs =',length(log.ohnolog_SV_notaffected_del)))

wilcox.test.del <- wilcox.test(sapply(dup.sum.list_for_del_filt, '[[', 2),
              ohnolog_noSV, 'less')

segments(x0=2, x1=3, y0=5.4)
segments(y0=5, y1=5.4, x0=2)
segments(y0=5, y1=5.4, x0=3)
text(x=2.5, y=5.7, labels = paste0('P = ', round(wilcox.test.del$p.value, 25)))
```

```
pdf('results/boxplot_expression_levels.pdf', width = 12, height = 8)


par(mfrow=c(1,2))
boxplot(list(SV.affected = log.ohnolog_SV_affected, 
             SV.not.affected = log.ohnolog_SV_notaffected,
             noSVoverlap = log.ohnolog_noSV), 
            col=c(alpha('darkred', 0.5), alpha('darkred', 0.25), 'white'), ylab='log10(sum tissue expression)', ylim=c(-2,6), main=paste0('ALL SV types\n', 'affected pairs=',length(log.ohnolog_SV_notaffected)))

wilcox.test.all <- wilcox.test(sapply(dup.sum.list, '[[', 2),
              ohnolog_noSV, 'less')

segments(x0=2, x1=3, y0=5.4)
segments(y0=5, y1=5.4, x0=2)
segments(y0=5, y1=5.4, x0=3)
text(x=2.5, y=5.7, labels = paste0('P = ', round(wilcox.test.all$p.value, 25)))


boxplot(list(SV.affected_del = log.ohnolog_SV_affected_del, 
             SV.not.affected_del = log.ohnolog_SV_notaffected_del,
             noSVoverlap = log.ohnolog_noSV), 
            col=c(alpha('darkred', 0.5), alpha('darkred', 0.25), 'white'), ylab='log10(sum tissue expression)', ylim=c(-2, 6), main=paste0('DEL SV types\n', 'affected pairs =',length(log.ohnolog_SV_notaffected_del)))

wilcox.test.del <- wilcox.test(sapply(dup.sum.list_for_del_filt, '[[', 2),
              ohnolog_noSV, 'less')

segments(x0=2, x1=3, y0=5.4)
segments(y0=5, y1=5.4, x0=2)
segments(y0=5, y1=5.4, x0=3)
text(x=2.5, y=5.7, labels = paste0('P = ', round(wilcox.test.del$p.value, 25)))


dev.off()
```

```
## quartz_off_screen 
##                 2
```

## SV overlap with ATAC

We find that SVs with high FSTs are enriched for putative effects on brain functions. We therefore tested if SVs also were enriched for potential effect on brain gene expression by affecting cis-regulator elements (defined as ATAC-peaks).

To do this we first identified genes with strong brain biased expression, since these are most likely to be impacted by selection pressure on cognitive abilities.

```
tissuepanel <- tissuepanel_raw

CV <- function(mean, sd){
      (sd/mean)*100
}

max.expression <- apply(tissuepanel[,-1], 1, function(i){
          dat <- i
          dat[is.na(i)] <- 0
          max(dat)>=1
      } 
      )

tissuepanel <- tissuepanel[which(max.expression),]


# brain must be top 3
brain_top <- apply(tissuepanel[,-1], 1, function(i) 1 %in% order(i, decreasing = T)[1:3])
tissuepanel <- tissuepanel[which(brain_top),]
```

We only considered genes that had an expression of >=1 TPM in minimum one tissue and where brain was among the tissues with top 3 highest expression level (23104 genes).

```
library(pheatmap)

pheatmap::pheatmap(tissuepanel[,-1], scale = 'row', cluster_rows = T, show_rownames = F)
```

Next we used an ATAC-peak data from brains of adult salmon to test if HIGH-FST SVs overlapped peaks (i.e. putative CREs) in highly brain biased genes more than expected by chance (all SVs used to test for FST outliers).

```
## Parsed with column specification:
## cols(
##   X1 = col_character(),
##   X2 = col_double(),
##   X3 = col_double(),
##   X4 = col_character(),
##   X5 = col_double(),
##   X6 = col_character(),
##   X7 = col_double(),
##   X8 = col_double(),
##   X9 = col_double(),
##   X10 = col_double()
## )
```

Out of a total of 14938 SVs, we found that 1149 SVs overlapped putative CREs (ATAC peaks) in genes (-3000bp downstream and +3000bp upstream genes).

In all furhter analyses, 12176 SVs which was used to test FST outliers between domesticated and wild salmon were used. Out of these 12176 SVs, 7.14% (869) overlapped a putative brain-CRE.

Only a slightly higher proportion of (8.48%, out of 566) overlapped brain CREs. To test if outlier FST-SVs were significantly enriched for SVs overlapping brain CREs we used a fisher exact test on counts.

```
# table : brain_ATAC in promoters  : is this comparison OK?

SVs_fst_ATAC <- SVs$FST[SVs$ATACpromoter %in% T] # table(SVs$ATACpromoter %in% T)
SVs_fst_NoATAC <- SVs$FST[SVs$ATACpromoter %in% F]

tab_promoters <- rbind(table(gsub('lier.*', '',SVs_fst_ATAC)),
                        table(gsub('lier.*', '', SVs_fst_NoATAC)))

rownames(tab_promoters) <- c('promoter_ATAC_SVs', 'SVs_no_ATAC')
test.tab.promoters <- fisher.test(tab_promoters)
tab_promoters
```

```
##                   Non-out Out
## promoter_ATAC_SVs     821  48
## SVs_no_ATAC         10789 518
```

```
# table brain_ATAC_brainexpressed in promoters

SVs_fst_ATAC_brainexpression <- SVs %>% filter(ATACpromoter) %>% filter(overlapgenes %in% tissuepanel$gene_id) %>% select(FST)

SVs_fst_NOT_atac_brainexpressed <- SVs %>% as_tibble() %>% filter(! FST %in% SVs_fst_ATAC_brainexpression$FST) %>%  select(FST)

# table : SVs ovelapping brain_ATAC in promoters  
tab_promoters_brainexpressed <- rbind(table(gsub('lier.*', '', SVs_fst_ATAC_brainexpression$FST)),
                                      table(gsub('lier.*', '', SVs_fst_NOT_atac_brainexpressed$FST)))

rownames(tab_promoters_brainexpressed) <- c('Brainexpressed_promoter_ATAC_SVs', 'Other_SVs')
test.tab.promoters_brainexpressed <- fisher.test(tab_promoters_brainexpressed)
tab_promoters_brainexpressed
```

```
##                                  Non-out Out
## Brainexpressed_promoter_ATAC_SVs     464  24
## Other_SVs                          11146 542
```

This test was not significant for outlier FST-SVs (p-value = 0.21), nor when subsetting for SVs (FST-outliers) overlapping CREs in genes with highly brain biased expression (N = 23104) (p-value = 0.74).

#### ATAC-SV overlap for outliers: FST-pvalue < = 0.05

```
SVs$pvalue <- SuppData6$`P-value`[match(SVs$FST, SuppData6$UniqueID)]
SVs_pvalue <- SVs[!SVs$pvalue %in% NA, ]


res <- SVs_pvalue %>% group_by(ATACpromoter, pvalue<=0.05) %>% summarize(res = n())

res <- matrix(res$res, nrow = 2, ncol = 2, byrow = T)
rownames(res) <- c('NoATAC', 'ATAC')
colnames(res) <- c('NoSig', 'Sig' )

test.tab.ATAC_FST <- fisher.test(res)

# SVs_pvalue %>% filter(pvalue>0.05)


SVs_pvalue_brain <- SVs_pvalue %>% filter(overlapgenes %in% tissuepanel$gene_id)

res_brain <- SVs_pvalue_brain %>% group_by(ATACpromoter, pvalue<=0.05) %>% summarize(res = n())

res_brain <- matrix(res_brain$res, nrow = 2, ncol = 2, byrow = T)
rownames(res_brain) <- c('NoATAC', 'ATAC')
colnames(res_brain) <- c('NoSig', 'Sig' )

test.tab.ATAC_FST_brain <- fisher.test(res_brain)
```

We asked if FST outliers (pvalue<=0.05, N=2233) were enriched in SVs overlapping brain ATAC peaks. Computer say “NO” (Fisher exact test, P=0.86). Same result was seen when only using ATAC peaks overlapping gene with very ‘brainy’ expression patterns (Fisher exact test, P=0.41).

```
sessionInfo()
```

```
## R version 3.6.3 (2020-02-29)
## Platform: x86_64-apple-darwin15.6.0 (64-bit)
## Running under: macOS Catalina 10.15.4
## 
## Matrix products: default
## BLAS:   /Library/Frameworks/R.framework/Versions/3.6/Resources/lib/libRblas.0.dylib
## LAPACK: /Library/Frameworks/R.framework/Versions/3.6/Resources/lib/libRlapack.dylib
## 
## locale:
## [1] en_US.UTF-8/en_US.UTF-8/en_US.UTF-8/C/en_US.UTF-8/en_US.UTF-8
## 
## attached base packages:
## [1] stats     graphics  grDevices utils     datasets  methods   base     
## 
## other attached packages:
##  [1] pheatmap_1.0.12    repmis_0.5         salmonfisher_0.1.1 forcats_0.5.0     
##  [5] stringr_1.4.0      dplyr_0.8.5        purrr_0.3.4        readr_1.3.1       
##  [9] tidyr_1.0.2        tibble_3.0.1       ggplot2_3.3.2      tidyverse_1.3.0   
## 
## loaded via a namespace (and not attached):
##  [1] Rcpp_1.0.4         lubridate_1.7.8    lattice_0.20-41    assertthat_0.2.1  
##  [5] digest_0.6.25      plyr_1.8.6         R6_2.4.1           cellranger_1.1.0  
##  [9] backports_1.1.8    reprex_0.3.0       RSQLite_2.2.0      evaluate_0.14     
## [13] httr_1.4.1         pillar_1.4.4       rlang_0.4.6        curl_4.3          
## [17] readxl_1.3.1       data.table_1.12.8  rstudioapi_0.11    blob_1.2.1        
## [21] R.utils_2.9.2      R.oo_1.23.0        rmarkdown_2.1      bit_1.1-15.2      
## [25] munsell_0.5.0      broom_0.5.6        compiler_3.6.3     modelr_0.1.6      
## [29] xfun_0.13          pkgconfig_2.0.3    htmltools_0.4.0    tidyselect_1.0.0  
## [33] fansi_0.4.1        crayon_1.3.4       dbplyr_1.4.3       withr_2.2.0       
## [37] R.methodsS3_1.8.0  grid_3.6.3         nlme_3.1-147       jsonlite_1.6.1    
## [41] gtable_0.3.0       lifecycle_0.2.0    DBI_1.1.0          magrittr_1.5      
## [45] scales_1.1.1       cli_2.0.2          stringi_1.4.6      farver_2.0.3      
## [49] fs_1.4.1           xml2_1.3.1         ellipsis_0.3.1     generics_0.0.2    
## [53] vctrs_0.3.1        RColorBrewer_1.1-2 tools_3.6.3        bit64_0.9-7       
## [57] R.cache_0.14.0     glue_1.4.1         hms_0.5.3          yaml_2.2.1        
## [61] colorspace_1.4-1   rvest_0.3.5        memoise_1.1.0      knitr_1.28        
## [65] haven_2.2.0
```
