# Supplementary material for: The structural variation landscape in 492 Atlantic salmon genomes
Source: Nat Commun. 2020 Oct 14;11:5176. doi: 10.1038/s41467-020-18972-x (PMC7560756; doi:10.1038/s41467-020-18972-x)
Supplement: Supplementary file 22 — Supplementary Data 19 [file 41467_2020_18972_MOESM22_ESM.zip › defining_duplicates-master/defining_duplicates.html]

Defining dulicates


Code 

- Show All Code
- Hide All Code

# Defining dulicates

#### Simen

#### spring 2020

- sub-tree splitting
- Gene tree topology based filtering and classification of rediploidization times
- the output: a ‘classification’ table of ss4r duplicates and singletons

## Defining Ss4R duplicates and singletons in the salmon genome

The pipeline for identifying a genome wide set of highly confident Ss4R duplicates can be described in the follwoing steps:

1. Synteny analyses was carried out using iadhore.
2. Orthogroup and gene tree estimation was carried out using the following pipeline:https://gitlab.com/sandve-lab/reeve/tree/master/data/from\_ortho\_pipeline
3. Each gene tree (based on CDS alignments) was split into smaller subtrees at the ‘N6’ node representing the divergence between pike and salmonids
4. For each sub-tree we then performed following filtering to extract Ss4R duplicates

   1. sub-trees must contain exactly 2 Atlantic salmon genes
   2. the putative Ss4R genes must be syntenic (i.e. for anchorpoints) from the iadhore analysis

### sub-tree splitting

```
spcTree <- read.tree("data/SpeciesTree_rooted_node_labels.txt")

plot(spcTree, use.edge.length = F,show.node.label = T)
```

Figure 1. Species tree with node names.

```
# make a table with the N6 nodes for each tree
OGtbl <- read_tsv("data/OGtbl.tsv",col_types = cols())

OG_Ntips <- OGtbl %>% group_by(OG) %>% summarise(length(unique(geneID))) # count the Nb genes/tips per orthogroup

# identify N6 nodes, and filter away NAs and redundancy
N6.nodes <- OGtbl %>% 
            group_by(OG) %>% 
            distinct(N6.nodes = as.numeric(gsub('.*\\.', '', N6))) %>% 
            drop_na(N6.nodes, N6.nodes)
            
           
N6.nodes$OG_Ntips <- OG_Ntips$`length(unique(geneID))`[match(N6.nodes$OG, OG_Ntips$OG)] # adds OG size collumn (nb tips per OG tree)

N6.nodes <- N6.nodes %>% filter(N6.nodes>OG_Ntips) # removed N6 nodes that are tip nodes (some weird artefact)


# get all CDS trees and 
allTrees <- readRDS("data/allTrees.RDS")
treeList <- lapply(allTrees, function(i) i@phylo)

     
clanList <- list()
for(i in 1:nrow(N6.nodes)){
  #print(i)
  #print(as.character(N6.nodes[i,2]))
  clanList[[i]] <- extract.clade(treeList[as.character(N6.nodes[i,2])][[1]], node = as.numeric(N6.nodes[i,1]))
  names(clanList)[i] <- paste0(as.character(N6.nodes[i,2]), '.', as.numeric(N6.nodes[i,1]))
}


# filter on clansize > 4
clanlist.nofilter <- length(clanList)
clanList <- clanList[sapply(clanList, Ntip)>4]
clanlist.sizefilter <- length(clanList)
removed.smallclans <- clanlist.nofilter-clanlist.sizefilter

# filter on salmo species -> clan need to contain 3 or more salmonids
salmonids <- c('Ssal', 'Omyk', 'Okis', 'Tthy', 'Hhuc', 'Salp')
fixnames = function(tipnames){
    gsub('.*\\_', '', tipnames)
  }

clanList <- clanList[sapply(clanList, function(i) sum(salmonids %in% fixnames(i$tip.label))>=3)]
clanlist.sizefilter.salmofilter <- length(clanList)
removed.toofewsalmospecies <- clanlist.sizefilter-clanlist.sizefilter.salmofilter

nb_ssal <- sapply(clanList, function(i) length(grep('_Ssa', i$tip.label)))
nb_splits <- table(substr(names(nb_ssal), 1, 11))
```

A total of 24475 CDS-tress from the orthogroup analyses was split on the N6 node (see figure 1) which resulted in 24618 sub-trees. Most (98.84%) of the orthogroup trees were split into 1-4 subtrees (Figure 2). Out of these subtrees 12922 trees (52.49%) had exactly two salmon genes and 8919 trees (36.23%) had one salmon gene.

```
barplot(table(nb_ssal))
```

Figure 2. Subtrees per orthogroup tree.

We then intersected the iadhore synteny information with sub-trees containing exactly 2 copies from Atlantic salmon (putative Ss4R duplicates).

```
anchorpoints <- read_tsv("data/OhnologTbl_Ssal.tsv")
```

```
## Parsed with column specification:
## cols(
##   multiplicon = col_double(),
##   OG = col_character(),
##   MRCA_Ortholog = col_character(),
##   Ds = col_double(),
##   gene.x = col_double(),
##   genome.x = col_character(),
##   list.x = col_character(),
##   coordinate.x = col_double(),
##   orientation.x = col_character(),
##   gene.y = col_double(),
##   genome.y = col_character(),
##   list.y = col_character(),
##   coordinate.y = col_double(),
##   orientation.y = col_character()
## )
```

```
ssal_inclans <- sapply(clanList, function(i) grep('_Ssa', i$tip.label, value = T))
ssal_2inclans <- ssal_inclans[sapply(ssal_inclans, length)==2]
ssal_1inclans <- ssal_inclans[sapply(ssal_inclans, length)==1]

DUPclantbl <- tibble(type='ss4r', clan = names(ssal_2inclans), gene1 = sapply(ssal_2inclans, '[[', 1), gene2 = sapply(ssal_2inclans, '[[', 2))


SINclantbl <- tibble(type='singleton', clan = names(ssal_1inclans), gene1 = sapply(ssal_1inclans, '[[', 1), gene2 = NA)

DUPSINtbl <- bind_rows(DUPclantbl, SINclantbl)

DUPSINtbl$duppairs <- apply(DUPSINtbl[,3:4], 1, function(i) paste(sort(gsub('_Ssal', '', i)), collapse='_'))

anchorpoints$duppairs <- apply(anchorpoints[,c(5,10)], 1, function(i) paste(sort(i), collapse='_'))

DUPSINtbl$is.syntenic <- DUPSINtbl$duppairs %in% anchorpoints$duppairs
```

Based on the raw output of the iadhore synteny block identification we found 15893 anchorpoints (i.e. potential ss4r duplicate pairs). Using the synteny relationships within the salmon genome (which can also contain synteny between older duplication events) we identified 10789 syntenic pairs of salmon duplicates from the sub-trees containing exactly 2 salmon genes (i.e. 83.49% of all putative ss4r in sub-trees).

### Gene tree topology based filtering and classification of rediploidization times

```
# if salmo monophyletic?
ss4r.clans <- DUPSINtbl %>% filter(is.syntenic) %>%
              select(clan) 

singleton.clans   <- DUPSINtbl %>% filter(type=='singleton', is.syntenic==F) %>%
                                   select(clan) 

ss4r.clansTrees = clanList[ss4r.clans$clan]
sing.clansTrees = clanList[singleton.clans$clan]


salmo.monophyletic=function(tr){
  trtest <- tr
  trtest$tip.label <- fixnames(tr$tip.label)
  is.monophyletic(trtest, tips = c(salmonids))
}

ss4r_salmomono <- sapply(ss4r.clansTrees, salmo.monophyletic)
ss4r_salmomono <- ss4r_salmomono[ss4r_salmomono]
sing_salmomono <- sapply(sing.clansTrees, salmo.monophyletic)
sing_salmomono <- sing_salmomono[sing_salmomono]

duptrees.mono <- length(ss4r_salmomono)
singletontree.mono <- length(sing_salmomono)

## filtering DUPSINGLtbl on monophyletic...

DUPSINtbl <- filter(DUPSINtbl, clan %in% c(names(ss4r_salmomono), names(sing_salmomono)))
```

To arrive at a ‘confident’ set of ss4r duplicates and singletons we finally perform gene tree topology based filtering steps.

**1. All salmon genes should form monophyletic clades with other salmonids AND clans should only contain a single pike ortholog**

We defined the salmonids as Ssal, Omyk, Okis, Tthy, Hhuc, Salp and used a small function to confirm salmonid monophyly:

```
salmo.monophyletic=function(tr){
  trtest <- tr
  trtest$tip.label <- fixnames(tr$tip.label)
  is.monophyletic(trtest, tips = c(salmonids))
}
```

Out of a total 10108 putative duplicate pairs, 10108 pairs also belonged to a gene tree which were monophyletic for the salmonids. For the singletons 8463 genes (total=8463 singletons) belonged to a gene tree which were monophyletic for the salmonids.

```
pike.orthos = sapply(clanList[DUPSINtbl$clan], function(i) {
  pikes <- length(grep('Eluc', i$tip.label))
  return(pikes)
  }
)


pike.0 <- as.numeric(table(unlist(pike.orthos))[1])
pike.1 <- as.numeric(table(unlist(pike.orthos))[2])

passed.filter <- names(pike.orthos)[which(unlist(pike.orthos)< 2)]

DUPSINtbl <- DUPSINtbl %>% filter(clan %in% passed.filter)
```

We then counted the number of pike genes pr clan. This is necessary for two reasons: \* Due to the nature of the species tree node-classification, clans with the several pike gene ‘outgroups’ can result in duplicated entries in the duplicate table (e.g. one clan with one pike and another with >1 pike genes). \* We want to only include a single pike ortholog in the table when it exists

After this filteringstep we are left with 18312 clans, containing 10023 duplicate paris and 8289 singletons.

```
DUPSINtbl$ID <- apply(data.frame(DUPSINtbl$gene1, DUPSINtbl$gene2), 1, function(i) paste(sort(i), collapse='_'))
dupl.entries <- DUPSINtbl$ID[which(duplicated(DUPSINtbl$ID))]
test.duplicatedenteries <- DUPSINtbl[DUPSINtbl$ID %in% dupl.entries, -6]
test.duplicatedenteries$clansize <- sapply(clanList[test.duplicatedenteries$clan], Ntip)
test.duplicatedenteries <- test.duplicatedenteries %>% arrange(clansize)
remove.duplicatedenteries <- test.duplicatedenteries$clan[duplicated(test.duplicatedenteries$ID)]

DUPSINtbl <- DUPSINtbl %>% filter(!clan %in% remove.duplicatedenteries)
sum.duplicatedenteries <- sum(duplicated(DUPSINtbl$ID))
```

Lastly we found 7 clans with size `n` that were subsets of other identical clans which included one additional tip (size `n+1`). We simply removed the largest of these duplicated clans. When we now check the table of dups and singletons we have 0 rows with duplicated entries of gene ID’s. *Thats great!*

Examples of such weird clans are:

```
par(mfrow=c(2,2))
plot(extract.clade(treeList['OG1v0003372'][[1]], node = 33), main='OG1v0003372.33')
plot(extract.clade(treeList['OG1v0003372'][[1]], node = 32), main='OG1v0003372.32')
plot(extract.clade(treeList['OG1v0003415'][[1]], node = 36), main='OG1v0003415.36')
plot(extract.clade(treeList['OG1v0003415'][[1]], node = 35), main='OG1v0003415.35')
```

**2. Classification of rediploidization timing of ss4r duplicates using topology-based filtering**

Due to the selection of genomes included in our gene trees we only classify rediplodization of ss4r into two categories - early or late (Figure 3).

```
par(mfrow=c(1,2))
plot(read.tree(text="(Eluc, (((Tthy, (Hhuc, (Ssal, (Salp, (Okis, Omyk))))), (Tthy, (Hhuc, (Ssal, (Salp, (Okis, Omyk))))))));"), main='Early rediploidization')
plot(read.tree(text="(Eluc, ((Tthy, Tthy),((Hhuc, (Ssal, (Salp, (Okis, Omyk)))), (Hhuc, (Ssal, (Salp, (Okis, Omyk)))))));"), main='Late rediploidization')
```

Figure 3. Two gene tree topologies used to classify ss4r into late or early rediploidization

This classification can however only be done on salmon genes that are contained in gene trees with two grayling genes (e.g. 7145, 66.22%).

```
# 2 tthy

tthy2 <- sapply(ss4r.clansTrees, function(i) length(grep('_Tthy', i$tip.label)))

ss4r.clansTrees_2tthy <- ss4r.clansTrees[tthy2==2]

Tthy_mono = sapply(ss4r.clansTrees_2tthy, function(i) is.monophyletic(i, grep('Tthy', fixnames(i$tip.label))))

LORe.clans <- names(Tthy_mono[Tthy_mono %in% TRUE])
AORe.clans <- names(Tthy_mono[Tthy_mono %in% FALSE])

DUPSINtbl$redip.class <- NA
DUPSINtbl$redip.class[as.numeric(na.omit(match(LORe.clans, DUPSINtbl$clan)))] <- "LORe"
DUPSINtbl$redip.class[as.numeric(na.omit(match(AORe.clans, DUPSINtbl$clan)))] <- "AORe"
```

Out of the 10023 remaining ss4r duplicate pairs passing all the above filtering steps are classified as having undergone 5657 ‘early’ rediploidization in an ancestor of all salmonids, while (1043) ohnologs are classified as having a ‘late’ rediploidization time, after the divergence of grayling/whitefish from the other salmonids.

### the output: a ‘classification’ table of ss4r duplicates and singletons

The results from this filtering pipeline is structured into a final table containing 18305 rows and 8 collumns. Each row represents either a duplicate pair (10023 rows) or a singleton gene (8282 rows).

```
DUPSINtbl <- select(DUPSINtbl, -c(is.syntenic, duppairs))
DUPSINtbl$gene1 <- gsub('_.*', '', DUPSINtbl$gene1)
DUPSINtbl$gene2 <- gsub('_.*', '', DUPSINtbl$gene2)

clanmatch = match(DUPSINtbl$clan, names(clanList))
clantrees.text <- c()
for(i in 1:nrow(DUPSINtbl)){
  clantrees.text[i] <- write.tree(clanList[[clanmatch[i]]])
}
    
DUPSINtbl$clantree <- clantrees.text
```

The resulting table is saved with the file name `table_Atlantic_salmon_dups_and_singletons` as both `.Rdata` and `.tsv` files.

```
save(DUPSINtbl,file ="data/table_Atlantic_salmon_dups_and_singletons.Rdata")

write_tsv(DUPSINtbl, "data/table_Atlantic_salmon_dups_and_singletons.tsv", col_names = T)
```
